# Ten simple rules for establishing a mentorship programme

**DOI:** 10.1371/journal.pcbi.1010015

**Published:** 2022-05-12

**Authors:** Anne M. Treasure, Siobhan Mackenzie Hall, Igor Lesko, Derek Moore, Malvika Sharan, Menno van Zaanen, Yo Yehudi, Anelda van der Walt

**Affiliations:** 1 Talarify, Kleinmond, South Africa; 2 Oxford Neural Interfacing, University of Oxford, Oxford, United Kingdom; 3 Open Education Global, Concord, Massachusetts, United States of America; 4 Weblearning, Johannesburg, South Africa; 5 Open Life Science, Wimblington, United Kingdom; 6 The Alan Turing Institute, London, United Kingdom; 7 South African Centre for Digital Language Resources, Potchefstroom, South Africa; 8 Department of Computer Science, University of Manchester, Manchester, United Kingdom

## Abstract

In recent years, a wide variety of mentorship programmes targeting issues that cannot be addressed through traditional teaching and learning methods alone have been developed. Mentoring plays significant roles in the growth and development of both mentors and mentees, and the positive impacts of mentoring have been well documented. Mentorship programmes are therefore increasingly being implemented in a wide variety of fields by organisations, academic institutes, businesses, and governments. While there is a growing body of literature on mentoring and mentorship programmes, gaining a clear overview of the field is often challenging. In this article, we therefore provide a concise summary of recommendations to consider when designing and establishing mentorship programmes. These recommendations are based on the collective knowledge and experiences of 4 different emerging and established mentorship programmes and can be adapted across various mentorship settings or contexts.

## Introduction

Mentoring has traditionally been defined as a method of professional and personal development where a person with expertise in a particular field or area of research (the mentor) advises and guides someone (the mentee) in that particular area or on specific skills [1–6]. Based on research over the past 20 years, the understanding of mentoring has evolved and it is now seen as a complex, collaborative, interactive process that may include more than 2 people [7]. Mentoring can be an effective approach for the development of knowledge and skills (e.g., [3]) and is formally used in a wide variety of fields, including, for example, science, technology, medicine, engineering, mathematics, education, business, and academia [2,6–14]. Mentor–mentee relationships play significant roles in the growth and development of both mentors and mentees, and the impacts of effective mentorship have been well documented (e.g., see [6,8]), including psychosocial [15,16], academic [2,3,7,8], and career [7,13,17,18] across diverse fields [7]. Furthermore, mentoring provides numerous benefits for both individuals and organisations [10], and mentorship programmes are increasingly being implemented by organisations, academic institutes, businesses, and governments [1].

Often mentoring occurs as a one-to-one relationship between mentor and mentee, but other types of mentoring are also used, such as peer mentoring [19,20], e-mentoring (also known as online mentoring, or telementoring) [1,21], group mentoring, or team mentoring (see [1,7] for more information). There is considerable variation in approaches to mentorship programmes [15], and those who establish such programmes need to make critical design choices. For example, much research has been done on the theory of mentorship, such as theoretical mentoring frameworks (see [6,7,15] for more information), which are useful for programme design [6,22–24]. In general, there has been a rapid increase in the available literature on mentoring, including mentorship programme tool kits, handbooks, and guides (e.g., [1,6,16,17]). This burgeoning literature is available in many areas including business mentorship, medical mentorship, youth mentorship, and, more recently, academic mentorship (as summarised by [8]). For each of these domains of mentorship, there are peer-reviewed publications of steps to build mentorship programmes and their effectiveness (e.g., general mentorship [18], reviews on youth mentorship [22], and medical mentorship [23]). However, with this complexity and breadth of mentorship literature, simplified summaries that can be adapted across various mentorship contexts are less common. In particular, guidelines based on the valuable lessons learnt from more than one mentorship programme appear to be limited.

Here, we present a concise summary of recommendations that outline key elements to consider when designing and establishing mentorship programmes. These recommendations are drawn from the collective experiences of 4 different emerging and established mentorship programmes as well as the outcomes of an online mentorship workshop held in April 2021 [24]. The mentorship programmes that contributed to this article include ESCALATOR Digital Champions Initiative (https://escalator.sadilar.org/champions/overview), Deep Learning Indaba (DLI) Mentorship Programme (https://deeplearningindaba.com/mentorship), Open Education For A Better World (OE4BW) (https://oe4bw.org; see [11]), and Open Life Science (OLS) (https://openlifesci.org). These 4 programmes vary in some of their particulars (age, size, target audience, focus subject areas, and duration of formal mentoring relationship), but overlaps occur, for example, in some common subject areas and target audiences (Table 1). In this article, we draw on the strength of these similarities and differences between the 4 programmes to provide an informed and summarised outlook on mentorship programme design and implementation based on the experiences and knowledge gained from each of the programmes.

**Table 1:** Details of the 4 mentorship programmes involved in this article.

	ESCALATOR	DLI	OE4BW	OLS
**Age**	Launched May 2021	Pilot June to December 2020; full-scale launch January 2021	Launched 2018	Launched 2019
**Size (number of mentors and mentees)**	6 tracks; number of participants accommodated varies	172 completed matches since full-scale launch in January 2021 (as of 22 November 2021)	2018–14 mentees; 27 mentors 2019–35 mentees; 40 mentors 2020–80 mentees; 74 mentors 2021–100 + mentees; 80 mentors	OLS1 (2020)– 29 mentees; 20 mentors OLS2 (2020)– 52 mentees; 36 mentors OLS3 (2021)– 66 mentees; 34 mentors OLS4 (2021)– 34 mentees; 36 mentors
**Target audience**	The programme is open to researchers, professional staff, and students from the 26 public universities and research councils in South Africa	African machine learning community members; participants range from all levels, including undergraduate students, research students, lecturers and academics, industry professionals, startups, and policy developers	All stakeholders worldwide, such as educators, practitioners or researchers, with an interest to develop OER on topics addressing 1 or more UN SDGs	Mentees are open-science curious researchers, students, and nonacademics who are interested in contributing to open research projects and communities. In this programme, they are supported by the organisers, mentors, experts, and other mentees in getting started with their journey as open research ambassadors
**Subject areas of focus**	Digital scholarship in the humanities and social sciences; open education	Machine learning, AI, and computational neuroscience	Supports the development and implementation of OER on topics with social impact according to the UN SDGs	Originally life sciences and bioinformatics, but quickly expanded to any research-related domain, including linguistics, anthropology, archaeology, robotics, machine learning, citizen and participatory science, open hardware, training, physics, and many more
**Duration of formal mentoring relationship**	6 tracks of varying lengths, from a few hours to 1 year	Strictly short term: typically 1 hour for a 1:1 meeting, with a possible 30-minute follow-up	6 months	16 weeks

AI, artificial intelligence; DLI, Deep Learning Indaba; OE4BW, Open Education For A Better World; OER, Open Educational Resources; OLS, Open Life Science; SDG, Sustainable Development Goal; UN, United Nations.

**Fig 1:**
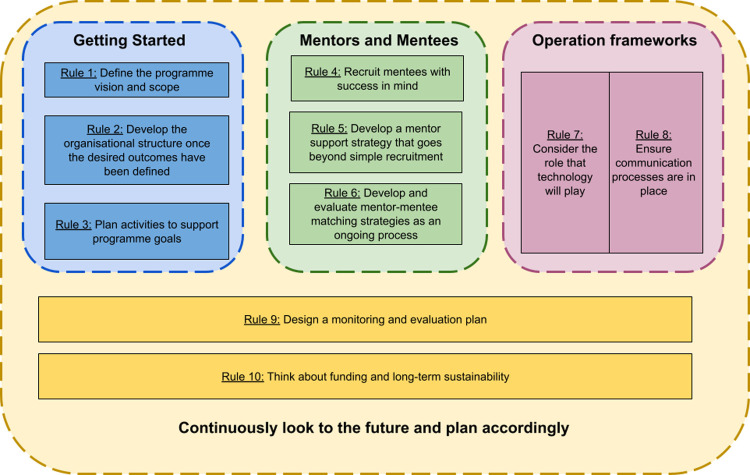
An overview of the rules and the relationships between them. The key considerations for establishing a mentorship programme can broadly be grouped into 3 categories, namely aspects necessary to consider when designing a mentorship programme; mentor and mentee topics; and operation frameworks. These considerations all rely on a crucial understanding of the long-term sustainability of the programme. The rules should not be viewed as sequential or linear as there is a large amount of interconnectivity between them.

The aim of this article is to outline the aspects necessary to consider when starting out with the design of a mentorship programme (Rules 1 to 3); mentor and mentee considerations (Rules 4 to 6); and, operation frameworks (Rules 7 and 8). The key elements in these broad categories all rely on an understanding of the long-term sustainability of the programme (Rules 9 and 10; see Fig 1 for a schematic overview of the rules and their relationships). These rules should not be seen as needing to be followed sequentially, and there is a large amount of interconnectivity between them. In order to demonstrate applications of the rules, for each rule, we provide a brief case study from one or more of the programmes involved in this article as examples. As all 4 programmes vary in some of their specifics (Table 1), we provide more in depth information about each of the programmes as they relate to each rule in S1 Text.

## Rule 1: Define the programme vision and scope

A clear vision and well-defined scope form the foundation upon which the rest of the mentorship programme is built and influences all other rules described in this article (for example, in [25], see Appendix A and Chapter 2). Spending more time early on in the design of the programme to define the “why” will save much time in identifying the “who,” “when,” “what,” “where,” and “how” discussed later on in this article.

The most essential component in this process is the identification of the need that is being addressed [1,16,17]. The programme organisers (see organisational structure in Rule 2) should have a shared understanding of the challenges and opportunities that are creating the need for mentorship, and a unified vision of the intended impact and expected outcomes should be developed. Outcomes should be clearly defined (see [1,6,7] for more information) and can include, for example, whether the programme seeks to create awareness, build knowledge or skills, modify practices, or lead to the development of a product. Knowing and specifying outcomes is instrumental in, for example, determining the optimal duration of each iteration of the programme, as well as the amount and intensity of the mentoring relationships (see [15] for details). Note that several iterations may be necessary to establish good workflows and to grow a community of mentors.

A programme’s vision and scope informs all aspects of a programme’s design, including deciding on the target audience and subject areas of focus (see DLI case study below), the recruitment of appropriate and committed mentees and mentors, as well as mentee–mentor matching strategies (discussed in Rule 6). For example, the expectations of both mentees and mentors should align with the programme’s vision. Importantly, monitoring and evaluation (M&E) strategies (discussed in Rule 9) should be implemented from the start of a programme to allow for an assessment of whether a programme is effectively aligning to its vision.

### Case study

The vision and scope of the programmes involved in this article largely revolve around their subject areas of focus (Table 1 and S1 Text). For example, the vision and scope of the DLI mentorship programme are based on the mission of the overarching organisation—to strengthen machine learning and artificial intelligence (AI) in Africa (see S1 Text). The DLI organisation works towards the goal of Africans being not only observers and receivers of the ongoing advances in AI, but also active shapers and owners of these technological advances (https://deeplearningindaba.com/about/our-mission). The DLI mentorship programme was explicitly designed to support this—strengthening machine learning in Africa by empowering the community. The outcomes of the DLI mentorship programme include the development of fundamental skills such as transforming a research idea into an experiment, writing a research paper, writing a research proposal, preparing a curriculum vitae, preparing a presentation or poster, preparing a postgraduate/internship/job application, preparing for an interview, and planning a career. The target audience is Africans who are active in the African machine learning community, and participants range from all career stages, including undergraduate and postgraduate students, lecturers and researchers, industry professionals, startups, and policy developers, in the fields of machine learning, AI, and computational neuroscience (see Table 1).

Thus, both the desired outcomes and the target audience are aligned with the vision and scope of DLI. DLI ensures that the recruitment of appropriate mentees and mentors is informed by the requirements and needs of the vision and scope, with expectations of prior knowledge in the disciplines mentioned above. The optimal duration for the formal mentoring relationship and the activities conducted during this time have both been designed by taking the desired outcomes (e.g., learning how to prepare a curriculum vitae) and the experience of the target audience into consideration. The duration is short term: a one-to-one meeting lasting 1 hour between mentor and mentee (with a possible follow-up), with the mentee needing to prepare beforehand.

## Rule 2: Develop the organisational structure once the desired outcomes have been defined

The organisational structure of the mentorship programme is built upon the programme’s scope and outcomes. Rolling out a successful, inclusive, and constructive programme requires input and contribution from a wide range of people [1]. Different roles and responsibilities need to be determined and fulfilled to provide administrative and advisory assistance and to ensure that stakeholders (funders, mentors, and mentees) are adequately supported and engaged. Such a foundational structure will facilitate and drive the development of the programme and facilitate the execution of the rules outlined in this article. Recruitment methods for people to fill organisational roles as well as term lengths for these roles can vary depending on the needs of the programme, and, often, these strategies are adjusted as the programme learns what works and what does not, while ensuring inclusivity (for example, see the case studies below).

The programme’s success relies on the organisational roles being founded upon a culture of accountability and openness. The following roles are proposed: programme directors (visionaries and administrators), programme coordinators (administrators), advisory board (visionaries), and, of course, the mentors and mentees. These roles are outlined in detail in [1] and have been implemented successfully in several of the programmes that have contributed towards this article.

The organisational structure will largely depend on the programme’s age and its unique vision and growth ambitions (which are likely to evolve over time). Some team members may occupy more than one role, and a programme might not need all the roles proposed in this rule to be filled from the beginning. Responsibilities associated with the different roles will often be time consuming and demanding. If possible, an incentive structure should be implemented to support long-term commitment. Establishing short-, medium-, and long-term goals early on will provide an outline as to when certain roles should be filled. A clearly defined organisational structure will also facilitate smoother transition and ensure coherence in the programme’s operationalisation should a core team member leave.

### Case study

A programme’s organisational structure will often depend on its age. For example, the organisational structure of ESCALATOR, a relatively young programme, is small. ESCALATOR is the only programme described in this article designed and implemented as part of a service provider agreement. Therefore, the management team consists of members from the client, the South African Centre for Digital Language Resources (SADiLaR) (https://www.sadilar.org) and the service provider Talarify (https://www.talarify.co.za). The programme is funded as a multiyear initiative through SADiLaR, one of the South African Research Infrastructure Roadmap entities. These entities form part of a larger strategic drive by the South African Department for Science and Innovation. The ESCALATOR management team meets once a week to discuss progress. Feedback is provided quarterly to the SADiLaR Steering Committee and the Audit, Risk and Finance Committee (https://www.sadilar.org/index.php/en/about/governance). No additional explicit or formal structure has been established within the ESCALATOR programme, although it is envisioned that this will emerge as the programme grows. Other people from SADiLaR and Talarify are invited as the need arises to participate in various roles, such as identifying requirements, providing feedback, and cocreating activities.

For a more mature programme, OE4BW, the organisational structure was also small during the first year of its implementation, only consisting of its initiators. As interest in the programme grew, the organisational structure evolved to introduce hub coordinators and the advisory board. Hub coordinators help manage processes, such as conducting regular follow-ups on progress with projects and monitoring mentor–mentee dynamics for projects allocated to their topical or geographical hub (https://oe4bw.org/projects). Advisory board members (https://oe4bw.org/advisory-board) provide strategic guidance to the programme’s organisers. Similar trends are seen in other programmes as well (see S1 Text).

The recruitment strategies used to fill organisational roles for a mentorship programme can vary. Often, these strategies will evolve as the programme matures. For example, during the first 4 years of the OE4BW programme (2018 to 2021), hub coordinators were identified and approached directly by the programme management. However, to expand the pool of hub coordinators and to give others opportunities to perform these roles and ensure greater diversity, from 2021, OE4BW includes a call for hub coordinators as part of the yearly application process for mentors and project leaders. Although hub coordinators are volunteers, they need to commit for a period of 6 months. There is no expectation of continuing in this capacity in the following year(s). OE4BW advertises the call for hub coordinators through social media channels (Facebook, Twitter, and LinkedIn) and various mailing lists, methods that have been successful for that specific programme. OE4BW advisory board members are identified and approached through recommendations. The board is quite diverse in terms of geographical representation, gender, and race. Members serve voluntarily and are expected to commit for 1 year but can step down at any time.

## Rule 3: Plan activities to support programme goals

Programme support processes should be designed to enable interactions between mentors and mentees, provide equitable access to resources and aid in monitoring progress. Embedding accessibility in these processes is important to ensure benefits for everyone irrespective of differing contexts (such as languages, cultures, demography, and social backgrounds), abilities, or disabilities (see Rule 8). With this in mind, and for the programme to effectively achieve its intended outcomes [15], a set of activities should be designed to support effective mentor–mentee engagements. These activities can supplement mentor–mentee interactions and should provide further opportunities for professional development for both mentors and mentees (see [7,26] for a discussion on the growth and development of mentors).

Activities work well if the expectations that surround them are discussed at the outset. The selection and sequence of activities will depend on the target audience and the contribution of the activities towards desired outcomes. These activities could either be structured or unstructured [15]. Programme organisers can set a curriculum [1] (e.g., https://content.prereview.org/openreviewers), or, alternatively, mentors may choose or design appropriate activities. The mentor and the programme organisers can use performance tasks to evaluate progress and measure impact (see Rule 9).

Activities should be designed to support uniqueness and growth, and to allow for both independent tasks and tasks that require interaction or assistance. Such differentiation will avoid spoon-feeding, encourage independent thinking, and foster conversations between mentor and mentee. The activities also need to be designed or structured to encourage performance and reflection. Mentorship programmes that rely exclusively on remote interactions should carefully consider which activities will support the development of a positive and constructive relationship between a mentor and mentee, and appropriate technology choices need to be made to facilitate this (discussed in Rule 7). Programme activities could also include growth opportunities for mentors to improve their mentoring skills (see [7,26] and the OLS case study in Rule 5).

The range of possible activities is vast, with many examples in the literature (e.g., see [1,7,15,16] and specific references provided below). Also refer to S1 Text and the case study below. For example, some programmes may use self-paced online education as part of the training (e.g., recorded webinars and videos using playlists such as ESCALATOR (https://www.youtube.com/channel/UCcLjjFCG2SNzZhxUJZyLcLg/playlists) and iBiology (https://www.ibiology.org/playlists/mentoring-science-trainees) or as a complete course (see https://ctsi.umn.edu/training/mentors/mentor-training). Such online learning resources can supplement real-time interactive mentoring and aid in issues around interactive mentorship education such as scheduling difficulties or other professional responsibilities of the mentors [7].

For programmes that aim to develop fundamental skills of mentees (e.g., DLI), activities could include how to write a curriculum vitae or scientific paper or how to prepare for an interview. In this case, mentees could be asked to read documentation before writing a draft of the discussion item in preparation for a meeting with the mentor. Mentees could be required to attend “hacking events” [27], conferences, seminars or workshops, or semester courses if at an academic institute [7]. Training could also include giving a presentation and receiving feedback to learn skills in listening, communication, and openness (for example, see the Sample Training Activity in Appendix I of [25]). The CORE Africa Research Mentorship Scheme (CARMS) programme is flexible and mentors use a “what works best” approach to support mentee development. Activities range from verbal advice during one-to-one meetings or phone calls, to online and in person lectures and presentations, as well as help with documents, and supplementary learning materials being provided to mentees [3].

### Case study

DLI aims to strengthen the African machine learning community via the development of fundamental skills. The programme matches community members with mentors for short-term personalised interactions across a wide range of topic areas (see the case study in Rule 1 for details). The programme includes one-to-one transactional interactions where mentees and mentors are provided guidelines to prepare. Activities can consist of watching tutorial videos, reviewing tutorials, and preparing some kind of tangible piece of work for the mentor to review. Mentees are expected to have at least a partial draft of the topic under discussion (e.g., research proposal, presentation, etc.). Resources are provided to guide mentees from any stage of progress. These are openly available online. See the mentor preparation document (https://docs.google.com/presentation/d/1oVFJPc4SKoxjWO62rwYZnL9h5SIDHxIKjxhakG5VXpQ/present?slide=id.g8e46e23741_3_8) and the mentee preparation document (https://docs.google.com/presentation/d/1oMP4YNNS-JwgKb7YhpWIC8NUj0QajGON-hEFQTtrRUE/present?slide=id.g8e46e23741_3_8). These guides can be adopted and adapted by other programmes. DLI ran 2 pilot programmes and focus groups to design the activities and supporting documents.

## Rule 4: Recruit mentees with success in mind

The mentee recruitment and selection processes require careful consideration to attract and select appropriate and committed mentees [3,6,16]. These considerations influence the mentors’ chances of affecting change, the mentees’ retention rates, and the ultimate success of the mentorship programme. Mentees’ expectations and selection practices should align with the programme vision (see Rule 1). The following outlines proposed factors that programmes can consider in mentees’ recruitment and selection strategies.

Advertising strategies to reach potentially suitable mentees will depend on the programme’s target audience. For example, issuing broad calls for mentees on social media may be less appropriate when trying to reach a specific community. In this situation, direct and personal invitations with the assistance of community leaders will work better.

Establishing a set of entry requirements is necessary to assess a mentee’s suitability according to the programme goals. The programmes involved in this article have adopted different approaches ranging from very minimal requirements to completing training courses or study resources as prerequisites for eligibility. Carefully designed programme application forms will help assess attributes and eligibility of potential mentees relevant to the programme (for example, see the OLS application form templates: https://github.com/open-life-science/application-forms). A good idea is to involve mentors in the mentee recruitment and selection process and to familiarise them with the applicants. Applications should be assessed according to a review rubric such as the one designed by OLS (https://github.com/open-life-science/application-forms/blob/master/review-rubrics.md) to assist with transparency, consistency, and fairness. The information that is solicited during the application process can include details about a mentee’s motivation for joining the programme, availability, ability to commit for the duration of the programme, and the type of support a mentee may require. While the programmes involved in this article use online application forms to assess mentees’ suitability, other forms of assessment or screening could also be adopted, such as interviews, references, consultations with the person who referred or nominated the mentee, or orientation sessions (e.g., see https://www.mentoringgirls.ca/recruitment-screening/screening-mentees).

The effectiveness of the recruitment and selection process should be measured by monitoring and evaluating interactions and experiences of both mentees and mentors, to ensure appropriate mentees are being selected (see Rule 9 for further discussion on M&E).

Digital privacy of all participants must be ensured and compliance with data protection laws is crucial (for example, the European Union General Data Protection Regulation (GDPR), the Protection of Personal Information Act (POPIA) in South Africa, etc.). A programme may need to comply with multiple data protection laws. It is therefore important that programme organisers familiarise themselves with regulations that may apply in regions where the programme will operate. For the programmes involved in this article, for example, DLI, OLS, and OE4BW comply with GDPR and ESCALATOR will comply with POPIA.

The number of mentees that the programme can support will depend on the resource limitations of the programme organisers responsible for its implementation. Accepting a large pool of participants can support growth ambitions but may be done at the expense of quality. Growth ambitions may also necessitate the deployment of additional resources to assist with the timely processing and assessment of applications across defined time periods. Given that programmes may end up with a large pool of prospective mentees, it might not be possible or even desirable to accept every eligible mentee that applied to the programme. In such instances, programmes should consider referring certain applicants to other mentorship programmes or sharing resources for additional support.

### Case study

OE4BW advertises a call for mentees (as well as mentors and hub coordinators) through social media channels (Facebook, Twitter, and LinkedIn) and various mailing lists. The OE4BW advisory board also plays a role in active promotion. The programme uses carefully designed application forms to assess the attributes and eligibility of potential mentees. Prospective mentees need to provide detailed information about their proposed projects, including a project plan, and their motivation for joining the programme (see application form in S2 Text or using this link: https://oe4bw.org/application-form-developers). The mentees’ open education resources projects are chosen based on their social impact, maturity of the idea for the resource, and estimation of the project feasibility. Furthermore, the projects need to align with one of the United Nations Sustainable Development Goals (SDGs), which is a paramount part of the programme’s vision (see S1 Text and Rule 1).

Notably, the selection process for mentees can develop over time. For example, because the Digital Humanities and Computational Social Sciences communities in South Africa are still relatively small and somewhat fragmented, the first part of the ESCALATOR programme has been about growing awareness and getting to know the community. In Phase I, only “light versions” were launched for 2 of the 6 proposed mentorship tracks. The “light versions” did not include recruitment of mentees for structured pairing with mentors. Instead, the tracks focused on awareness creation events to introduce the concept of mentorship and show the benefits of joining a community of practice. The first track had no minimum requirements for entry, and a selection process was not implemented. The registration form for this track is available online (see S3 Text). A more structured approach is being taken for a third track launched in 2022. Here, mentees have to submit proposals detailing what they would like to achieve by joining the mentorship programme and what they expect from the programme team. Some of the questions in the application form were adopted from the OLS programme application form (see S4 Text). Applications will be evaluated by a selection committee. See S1 Text for more information on ESCALATOR’s proposed mentorship tracks.

## Rule 5: Develop a mentor support strategy that goes beyond simple recruitment

When establishing a mentorship programme, it may be tempting to select mentors based solely on their experience or competency in their area of expertise. It is, however, equally important to consider mentors’ interpersonal skills, sensitivity to different mentees’ contexts and their capacity to support a mentee to be successful in the programme. In addition, mentor interest and motivation are important predictors of effective mentoring [2], and mentor commitment and programme understanding are crucial to a programme’s success [28].

Programme organisers have to decide on a mentor recruitment strategy. For example, targeted recruitment, which has proven effective, versus screening processes (see [29] for more details). A programme could also design and implement a marketing strategy (e.g., see [29,30] for more information). Screening methods can include online application forms (see the OE4BW form: https://oe4bw.org/application-form-mentors), formal “job” application processes, interviews, and references to assess the suitability of prospective mentors (e.g., [3,29]).

Recruited mentors are likely to exhibit a range of experience and expectations. Mentors will require different levels of support and capacity development as advanced expertise does not always directly translate to the ability to communicate with and guide a mentee productively. Therefore, the professional development of mentors should not be ignored [6,7,16,26], and, as discussed in Rule 3, activities should be designed to promote mentor growth and development. Furthermore, co-mentoring of a mentee provides mentors with the opportunity to learn from each other and could be considered as part of the mentor–mentee matching strategy (elaborated on in Rule 6). Mentor support strategies can take on multiple forms and are especially dependent on the typical duration of the mentor–mentee interactions (see Rule 6) and the experience and needs of the participating mentors. Based on our collective experience, we propose a few strategies to ensure mentors remain committed and contribute to achieving a programme’s goals.

Onboarding can help to align mentors to the programme’s mission, vision, and expectations and can provide understanding of the specific needs of the mentees. Onboarding procedures can be made available as documents, videos, training sessions, and/or meetings between programme organisers and mentors. For example, see the onboarding document provided to mentors for the DLI programme (https://docs.google.com/presentation/d/1oVFJPc4SKoxjWO62rwYZnL9h5SIDHxIKjxhakG5VXpQ/present?slide=id.g8e46e23741_3_8).

Clearly defined communication channels for mentors to contact programme organisers are essential to make them feel comfortable reporting potential concerns and have faith that the programme team will respond appropriately. Continuous communication and feedback are equally important in ensuring that mentors feel valued and appreciated for their contributions to mentees and the programme’s goals.

Importantly, support should include mindfulness of the time commitment and investment on the part of the mentors: A culture of flexibility and understanding can aid in long-term commitment and avoid mentor burnout. Adequately trained and supported mentors also lay the foundation for successful mentor–mentee interactions, elaborated upon in Rule 6. Furthermore, as mentioned above and in Rule 9, mentor experiences and interactions with the mentees and the programme should be evaluated using M&E strategies. These evaluations will help improve mentors’ participation in the programme by bringing to light successes and challenges faced by mentors.

### Case study

As ESCALATOR is a young programme, the initial phase focused on growing awareness and getting to know the community while learning about availability of potential mentors for the various proposed mentorship tracks. No external mentors have been recruited, and the programme management team has taken on mentorship roles where required. As community members join activities and communication platforms (e.g., Slack), spontaneous peer mentoring has started to emerge, which is highly encouraging. More formal mentor support strategies will need to be developed for future tracks with more structured formats (see S1 Text for more information).

OLS, which is comparatively more mature, has an established strategy for recruiting and supporting mentors. OLS involves open science practitioners who provide real-world examples and help integrate contextualised knowledge to design and lead open research in local communities. They are on boarded as mentors, experts, and advisors in the programme and provided with a comprehensive overview of available resources. Professional mentorship and coaching training forms part of the onboarding process. Graduates from the previous rounds are invited as mentors and provided a co-mentor for support if required. A clear opportunity for offboarding or leaving the programme allows mentors to take a break when needed. Due to external funding, honoraria are offered to mentors to recognise and recompense them for their time and investment in the programme.

## Rule 6: Develop and evaluate mentor–mentee matching strategies as an ongoing process

Careful consideration of the initial mentor–mentee match and continuous monitoring of the match dynamics and productivity are essential to ensure effective mentor–mentee interactions [6,16].

The exact criteria for matching a mentor to a mentee are critical and should be informed by the programme goals [16]. Matches are influenced by the duration of the mentor–mentee interactions and the target audience. Considerations include the mentor’s expertise and experience and how this aligns with the mentee’s expectations and needs (e.g., [3]). Factors such as differences in race, gender, language, and geographical contexts (for example, locations, time zones, and cultures) should be considered to ensure inclusivity and accessibility. These differences also aid in bringing cross-cultural perspectives, which are often appreciated by mentees [7,11] and mentors. Some mentees may require additional considerations and tailored interventions according to their unique circumstances and preferences. Geopolitical contexts, cultural specificities, or infrastructural challenges may have implications for both mentor and mentee participation, mentor–mentee dynamics, and collaboration [16]. Mentors and programme organisers may need to invest more time and adapt their approaches to address unique circumstances.

Mentorship programmes can adopt different mentor–mentee matching approaches [31]. As a collective experience, the programmes that have contributed to this article have found a human in the loop essential in ensuring positive and productive matches. Mentors are typically included in the final decision, having assessed the mentee’s application themselves. Fully automated tools are avoided as these may lose the nuanced consideration necessary for successful matches.

Mentor–mentee matching is an ongoing process that extends beyond the initial match. Continuous M&E of interactions and experiences of mentors and mentees as well as safe feedback protocols need to be in place. These should be managed by the programme organisers to ensure that matches are suitable and mentor–mentee dynamics are effective [1,7]. Feedback strategies and M&E are discussed further in Rule 9. Despite due diligence, sometimes an inappropriate match is made, and procedures need to be in place to address concerns and to replace mentors. A mentee’s needs may also evolve over time, which may necessitate introducing a new mentor or a co-mentor with additional skills and expertise. Co-mentoring provides an opportunity for mentors to learn from each other.

The duration of the formal mentoring relationship depends on the structure of the mentorship programme, the programme’s vision and scope (described in Rule 1), and the programme’s goals. For example, mentorship guidance required for writing a curriculum vitae would be less than what is needed for developing a new tool or community. For the mentorship programmes involved in this article, mentoring relationships range from a few hours to a year (see Table 1).

Expectations should be communicated upfront to mentors and mentees [16,17], and there should be no obligation for a mentor to support a mentee beyond the programme requirements. Should a mentor wish to continue staying in touch with a mentee, this is of their own accord, and the mentorship programme should not be held responsible for continued engagement. Clear expectations of time commitments should be laid out and a formal document such as a Memorandum of Understanding could be used for this. There could also be an opportunity for mentees to become involved in the mentorship programme by becoming mentors themselves if they fit the required skills and adhere to the requirements of the programme (e.g., see the OLS case study in Rule 5).

### Case study

The DLI mentorship programme requires prospective mentors to review onboarding information (https://docs.google.com/presentation/d/1oVFJPc4SKoxjWO62rwYZnL9h5SIDHxIKjxhakG5VXpQ/present?slide=id.g8e46e23741_3_8). Following this, mentors complete a sign-up form to specify their expertise and availability. This information is used to match them as effectively as possible with mentees (see https://deeplearningindaba.com/mentorship/mentor). Mentees are required to read preparation materials (https://docs.google.com/presentation/d/1oMP4YNNS-JwgKb7YhpWIC8NUj0QajGON-hEFQTtrRUE/present?slide=id.g8e46e23741_3_8) before completing an online application form at least 3 weeks before any deadline, to give mentors enough time to arrange their schedules. DLI reviews the applications and screens available mentors to assess whether an appropriate match can be made. Depending on mentors’ availability, a match may not be available within the required time frame. If an appropriate mentor is available, a mentee receives an email connecting the mentee with the mentor within 1 week of submitting the application form.

DLI is very explicit about the duration of the formal mentoring relationship, which is communicated upfront to both parties. A mentee can apply for a session in a specific mentorship area and can expect to be matched with an appropriate mentor within 1 week. The first meeting is a 1-hour video call, with the potential for one follow-up. Mentees may apply for multiple sessions in different mentorship areas. DLI provides documentation to help mentees prepare for the sessions. The expectations are clearly laid out (one call and one optional follow-up). Beyond that, the mentor is under no obligation to support the mentee.

DLI sends a feedback form to all mentees following interactions with the mentors (see the feedback form: https://docs.google.com/forms/d/e/1FAIpQLSefkBVH8HDzZkltnxhpp_3HJU1rtMeF9hvqNAm-OlJWMCMqJA/viewform). The form collects both text-based responses and scores. These responses are routinely reviewed. If a mentee scores a mentor poorly, all administrators of the programme are notified, and an automatic email is sent to the mentee to encourage the provision of more information and the opportunity to reapply for a different mentor. The same mentor–mentee match will not be made in future. There are open channels of communication and mentors and mentees are consistently encouraged to report any problems.

## Rule 7: Consider the role that technology will play

The technologies chosen will play a significant role in how participants can and will engage in the mentorship programme [6] and should be informed by the programme’s communication strategy (see Rule 8). Programme organisers must consider the digital literacy of participants when making choices and provide support for participants who are unfamiliar with specific tools. The following should be considered when setting up the programme’s digital infrastructure: geographical contexts and related restrictions, accessibility requirements for all participants, and available resources as well as financial or technical barriers to using them. A small selection of tools curated by careful testing can mitigate confusion, frustrations, and, importantly, digital exclusion (e.g., see [32]).

Technical aspects such as access to the internet or digital infrastructure can also impact participants’ overall experience. Tools that work well in one setting may not work well in another. Tooling should be tested with participants to establish whether it is accessible, if electricity and internet capabilities are sufficient, and whether additional support should be provided.

Finally, programme organisers may want to consider the value alignment of tools selected for the programme. For example, some organisations might prefer to self-host open-source technological solutions, whereas it might be more convenient for other organisations to pay for a proprietary solution instead to avoid the administrative burden of self-hosting. These decisions will also be affected by the available funding, administrative support, or size of the organisation to share the responsibility of infrastructure development, coordination, and maintenance.

### Case study

OLS uses various tools aligned with the requirements and accessibility of all participants (e.g., see [32]). Online training calls are delivered in English via Zoom that integrates with Otter.ai for live transcriptions. Live transcription is particularly crucial for people with low internet bandwidth, hearing accessibility needs, or those who do not use English as their primary language and prefer to follow audios along with written text. A collaborative document (using HackMD or Etherpad) with a clear agenda is set up for each call and facilitates communication of important information as well as shared note-taking. Calls are recorded and shared via YouTube for those who are unable to attend in real time. Self-paced learning is facilitated through paired training sessions with assignments to help mentees reflect on lessons learned from the respective calls. Participants use GitHub to record their progress and engage with each others’ projects. GitHub training is given at the beginning of the course to ensure everyone can use GitHub or host simple Git pages for their projects. The programme team uses a shared Google workspace to manage and store their resources and gather feedback from all participants in a centralised location. Training materials are shared via Google Drive and are cross-posted on Zenodo under open licenses. The choices of technology are handled case by case and depend on the tools used by a specific open science project, their user-friendliness, and availability across different geographic locations.

## Rule 8: Ensure communication processes are in place

In any mentorship programme, communication is a crucial aspect [1,6]. The programme organisers need to think about processes that will enable effective communication among all stakeholders in the programme. It is essential to develop communication methods and strategies that are inclusive of everyone in the programme [33]. The communication strategy thus impacts the technologies used (see Rule 7). A programme website, social media, or an online repository can be effectively used to share documentation, communicate ways of working, make announcements, and enable access to relevant information as needed. Communication tools selected for the programme should facilitate asynchronous interactions which is beneficial for both online and in-person programmes. For example, specific online tools designed for accessibility can be used for maintaining regular communication, sending updates, and holding mentor–mentee meetings (e.g., see [32] and Rule 7).

Accessibility criteria should be considered carefully for communication processes to ensure that all participants benefit, irrespective of differing circumstances, abilities or disabilities. Most notably, accessibility requirements related to the languages, cultures, demography, any disabilities, and social backgrounds of the participants should be kept in mind. Programme organisers should provide appropriate feedback mechanisms (M&E is discussed in Rule 9) that can allow participants to request adjustments to accommodate their participation-related requirements in the programme. All participants should be made aware of the programme’s policies that ensure a welcoming and safe space for collaboration, protects them against potential harm, recognises their achievements, and provides future opportunities for growth. The programme’s code of conduct should be easily accessible and included in the onboarding process. Additionally, a reporting procedure (for example, for grievances) should be put in place.

Although an effective strategy and pathways for communication can offer a platform to connect, experience gained through such connections can have a transformational impact beyond the programme. These communities provide opportunities for discussions and knowledge exchange within a supportive network even after the programme is over. However, as discussed above in Rule 6, a mentor’s obligations towards a mentee should not extend beyond the programme’s requirements and any communication that occurs after completing the programme is of the mentor’s and mentee’s own accord.

### Case study

OLS adopts a communication strategy that ensures inclusivity of all participants by taking accessibility requirements into account, making sure that information is readily and easily available, and facilitating synchronous and asynchronous operation modes. The OLS website is developed on GitHub and hosted at https://openlifesci.org. The website is used for open and transparent communication of different roles and opportunities in the programme. The OpenReview platform is used to receive and review applications, and to maintain communication with applicants. A community participation guideline, code of conduct, and list of responsibilities for all the participants are communicated clearly via the website and shared via emails. A team email address is shared in all relevant resources to allow participants to contact the programme team. Twitter and a public mailing list are used to announce new calls for applications and share resources online. Information is shared via an email from the programme team at the beginning of each week via a mailing list dedicated to the current cohort. Personal support is provided by maintaining communication via Slack channels and personal mentorship calls for check-ins are hosted using the online platform of the mentees’ choice. A shared calendar is used to communicate programme schedules across different time zones and training calls are hosted via Zoom.

## Rule 9: Design a M&E plan

Ongoing M&E of a programme are crucial for quality improvement and for ensuring an effective mentorship programme [1,7]. Decide in advance how the mentorship programme will be evaluated and how the impact of the programme will be demonstrated (e.g., see [1,34]). For example, a Theory of Change [35] and Logical Framework (logframe) [36] are tools that aid in staying focused on the project aims and being explicit about causal relationships between inputs, activities, outputs, and the outcomes. The Logical Framework Approach (https://www.betterevaluation.org/en/evaluation-options/logframe) is a well-known programme planning, monitoring, and evaluation methodology. A logframe matrix translates the Theory of Change into practice and forms the basis of an actionable, measurable plan for project implementation.

Participant surveys and other feedback processes such as one-to-one meetings or focus groups can be used to evaluate mentors’ and mentees’ initial expectations, progress, and experience through each iteration of the programme. Evaluation methods and information gathered will likely vary depending on the stakeholders and the timeline of the programme. Consider how information can be packaged for different audiences. For example, a mentor or mentee might assert that learning basic technical skills is a high-impact outcome, whereas a funding body might want to see a medium- or long-term impact that aligns with their strategic goals. Monitoring and evaluating mentee and mentor experiences is also vital to ensure successful mentor–mentee matching strategies as discussed in Rule 6 (and see [1,3]). M&E strategies also inform many other aspects of a mentorship programme, such as ensuring an effective organisational structure, activities, mentee and mentor recruitment, technology used, and communication processes (all discussed above). Making sure that a programme shows long-term sustainability, which may be essential for securing funding (see Rule 10), also relies on sound M&E strategies.

Consider data protection and privacy implications when collecting feedback or assessing individual participants or projects (discussed in Rule 4). Keep the evaluation metrics straightforward so that these can be easily adopted by different stakeholders in the programme. For example, both mentors and mentees can evaluate their progress by identifying their personal goals in the programme, defining where they are in their roadmap, what strategies or tools they used in the programme, and how that helped achieve their goals at the end of the programme. This method can be applied to also measure the overall impact of the programme. At the end of each iteration, individual and programme-wide strategies can be assessed to reflect on the success as well as changes that should be introduced in the future.

A good idea is to consider quality over quantity (such as the maximum number of mentors or mentees) in order to ensure effective resource allocation and sustainability in the programme. Finally, share the data in the form of postprogramme reports such as blogs (e.g., see https://escalator.sadilar.org/post), formal documentation [37], or peer-reviewed publications [11]. Such reporting showing the impact of the programme will help to build trust in the community, attract new participants or funding, and support stronger programmes within networks, thereby promoting long-term sustainability (see Rule 10).

### Case study

OE4BW: The first 2 programme iterations (2018 and 2019) were evaluated through feedback surveys for mentors and mentees (see S5 and S6 Texts). Following this evaluation process, several changes were made to the programme, including the introduction of hub coordinators to manage the programme scale. Hub coordinators provide regular feedback about the projects in different hubs to the programme organisers, and their feedback is also discussed during the advisory board meetings. Mentors and mentees have an opportunity to provide overall impressions about the programme during the final OE4BW event that usually takes place as an in-person meeting (2018 to 2019) or online (2020 to 2021). Evaluations using feedback surveys for mentors and mentees will also be conducted with the 2020 and 2021 cohorts.

Targeted surveys and feedback forms are used by DLI and OLS for both mentors and mentees. As part of a mentor–mentee matching strategy, DLI sends a feedback form to all mentees following interactions with the mentors (refer back to Rule 6 and its case study for more information and see the DLI feedback form in S7 Text). DLI continuously reviews the forms to ensure the programme is as accessible as possible and that the mentor–mentee interactions are productive. DLI management meets on a needs assessment basis to assess the programme processes and to suggest and implement changes.

OLS uses targeted surveys in the middle and at the end of each cycle to assess the success and impact of the programme (see S8 and S9 Texts for mentee surveys and S10 Text for mentor survey; available from: https://github.com/open-life-science/cohort-surveys). Online interactions and engagements are supported via Slack channels and mentors keep track of mentees’ progress through regular check-ins. A dedicated Slack channel for anonymous reporting and feedback is provided as an additional support option to participants. All activities are summarised and published as annual reports and feedback from the participants is integrated to revise the next iteration of the programme. A research project has been designed to conduct a long-term impact study to specifically report on training and mentoring practices in open science that are successful and transformative for local communities.

ESCALATOR developed a Theory of Change [35] and Logical Framework [36] as tools for M&E (see above). The management team performs a quarterly review of the programme. The report is shared with the Steering Committee and the Audit, Risk and Finance Committee for internal discussion and feedback. A more formal midterm review was executed at the end of December 2021 through a series of meetings to consider the programme’s status and the impact of the Coronavirus Disease 2019 (COVID-19) on planned activities. The outcome of these strategic meetings and the review informed a revision of the project plan, Theory of Change, Logical Framework, and reprioritisation of activities for 2022 and beyond. ESCALATOR uses a registration form (S3 Text) and a feedback form (S11 Text) to assess participation and evaluate effectiveness of one of the mentorship tracks available on the programme. Similar forms will be developed for each of this programme’s tracks to assess success and impact.

## Rule 10: Think about funding and long-term sustainability

Some mentorship programmes may start with large-scale funding for development, whereas others start small and scale up. For the latter, starting a programme with absolutely no funding or on a shoestring budget using free resources and volunteer labour is feasible. However, financial requirements are likely to arise sooner or later in the programme as it scales. Therefore, think about both the human resource and financial costs during the earlier stages of the programme. The budget and business plan for the programme should involve small achievable steps as well as a longer-term vision. Identify potential funding sources for small grants or sponsored services to reach short-term goals and larger funding or partnership options in the long term to sustain the demand for budgeted items in the programme. We consider the following example: an earlier stage of an online programme might involve infrastructure costs such as web hosting or teleconferencing facilities. In the later stages of the programme, the cost for programme organisers who can contribute to the development of the programme’s sustainable strategies could be considered. The output of M&E measures (see Rule 9) will be important to ensure that the programme achieves long-term sustainability.

If funding for the mentorship programme is not available from the onset, a proposal to raise funds through grant applications or sponsorships can be developed after the completion of the first iteration of the programme. Short-term budgetary considerations could include paid support for mentor time when possible and bursaries for mentees to enable participation. In the long term, resources would likely be needed for the programme to be self-sustaining with full-time employees. Finally, if funding is limited or time bound, make sure this is clearly communicated to programme participants and other stakeholders. Archive programme resources, preferably under open licensing where possible (for example, see https://creativecommons.org/choose), to allow others in the community to reuse them in the future. Openly publishing papers, documents, and reports to demonstrate programme success, and viability is also a good idea in order to indicate long-term sustainability and fundability of the programme (e.g., see [11,37]).

### Case study

In the initial phase in 2019, OLS relied fully on volunteer labour and small infrastructure support. By successfully piloting the programme and running a cohort with 20 international projects led by 29 members from 5 continents, OLS highlighted a demand for structured training and mentoring in Open Science. The viability of the project was demonstrated, the impact of the programme was assessed, and an annual report was openly published to share the lessons with the community (see [37]). In the subsequent phase, OLS worked towards maintaining the quality of the activities and acquired funding through independent funding schemes to support mentees’ participation and offer honoraria to mentors to recompense for their time. This period was used to build collaborations with international institutions and local communities and to pursue support from universities and funding bodies to make the programme more sustainable over the long term. In the third (current) phase, significant funding was acquired to hire members to support the sustainability of the programme. A formal research project will also be launched to measure and record the impact of training and mentoring in open science. For all of these steps, M&E has been crucial to track progress and indicate the need for the programme (see Rule 9).

## Conclusions

Mentorship programmes, where experienced mentors guide mentees interested in learning and obtaining growth in a particular area or field, can make a massive impact on the training and development of people. However, the effectiveness of these programmes depends heavily on their design and proper establishment. This article deals with aspects that require careful consideration when designing and implementing mentorship programmes. The points we discuss are a concise summary of recommendations based on the collective knowledge and experiences of the 4 mentorship programmes involved in this article and enriched by the outcomes of an online mentorship workshop held in April 2021 [24]. The rules should not be viewed as sequential or linear, and there is a large amount of interconnectivity between them.

At the outset, the programme’s vision and scope needs to be carefully considered (see Rule 1). Based on the scope, a range of desired outcomes and goals can be defined, which directly influences the organisational structure (see Rule 2). The vision and scope will also have an impact on all other considerations we describe, for example, activities (Rule 3), mentee (Rule 4) and mentor (Rule 5) recruitment, and mentee/mentor matching strategies (Rule 6).

The practical phase of the programme depends heavily on successfully matching mentors and mentees, the mentees’ characteristics (see Rule 4), and the mentors’ commitment and involvement (see Rule 5). Once the mentees are matched with the mentors, continuous monitoring of the collaboration will need to take place (see Rule 6). During the programme, a breadth of tools can be used to support projects’ technological needs (see Rule 7) as well as communication. It is important, however, to think beyond technology when designing the communication strategy (see Rule 8). To ensure an effective programme, evaluation and impact measures should be implemented from the very start of the programme (see Rule 9). Feedback from these M&E strategies is useful for informing various aspects of the programme, such as activities, mentee and mentor recruitment, mentee–mentor matching strategies, technology used, and communication processes. The output of M&E measures will also be useful for considering whether a programme’s organisational structure is effective and in making sure that the programme shows long-term sustainability for which funding may be essential (see Rule 10).

The rules presented in this article highlight important aspects that should be taken into account when establishing a mentorship programme. Considerations discussed are relevant to both small and large programmes within a wide range of application areas, and the pointers and examples given can be adapted as necessary.

## Supporting information

S1 Text Programme-specific information relating to each rule. In order to demonstrate applications of each of the rules, for each rule, a brief case study is provided from one or more of the programmes involved in this article as examples. However, as all 4 programmes vary in some of their specifics, more in depth information about each of the programmes as they relate to each rule is provided in this appendix table.
(PDF)

S2 Text OE4BW mentee application form. OE4BW uses carefully designed application forms to assess the attributes and eligibility of potential mentees to the programme. Prospective mentees need to provide detailed information about their proposed projects, including a project plan, and their motivation for joining the programme. The mentees’ open education resources projects are chosen based on their social impact, maturity of the idea for the course, and estimation of the project feasibility. Furthermore, the projects need to align with one of the UN SDGs—part of the programme’s vision and scope. OE4BW, Open Education for a Better World; SDG, Sustainable Development Goal; UN, United Nations.
(PDF)

S3 Text ESCALATOR EXPLORER track registration form. The registration form for one of the tracks of the ESCALATOR mentorship programme (EXPLORER). This track had no minimum requirements for entry, and a selection process was not implemented. The form was used to assess participation only.
(PDF)

S4 Text ESCALATOR EDUCATOR track application form 2022–2023. The application form for one of the tracks of the ESCALATOR mentorship programme (EDUCATOR) for 2022–2023. ESCALATOR implemented a more structured approach for this track as opposed to previous tracks, and mentees have to submit proposals detailing what they would like to achieve by joining the mentorship programme and what they expect from the programme team. Some of the questions in the application form were adopted from the OLS programme application form. Selection of mentees will be done by the programme team.
(PDF)

S5 Text OE4BW mentor follow-up questionnaire. The OE4BW follow-up questionnaire/feedback survey for mentors. The first 2 iterations of OE4BW (2018 and 2019) were evaluated through feedback surveys for mentors and mentees. Following this evaluation process, several changes were made to the programme, including the introduction of hub coordinators to manage the programme scale. Evaluations using feedback surveys for mentors and mentees will also be conducted with the 2020 and 2021 cohorts. OE4BW, Open Education for a Better World.
(PDF)

S6 Text OE4BW mentee follow-up questionnaire. The OE4BW follow-up questionnaire/feedback survey for mentees. The first 2 iterations of OE4BW (2018 and 2019) were evaluated through feedback surveys for mentors and mentees. Following this evaluation process, several changes were made to the programme, including the introduction of hub coordinators to manage the programme scale. Evaluations using feedback surveys for mentors and mentees will also be conducted with the 2020 and 2021 cohorts. OE4BW, Open Education for a Better World.
(PDF)

S7 Text DLI mentee feedback form. The DLI feedback form for mentees. As part of a mentor–mentee matching strategy, DLI sends a feedback form to all mentees following interactions with the mentors. DLI continuously reviews the forms to ensure the programme is as accessible as possible and that the mentor–mentee interactions are productive. DLI management meets bimonthly to assess the programme processes and to suggest and action any changes. DLI, Deep Learning Indaba.
(PDF)

S8 Text OLS post OLS-4 mentee survey form. The mentee feedback form sent to mentees at the end of the OLS-4 cohort for OLS. OLS uses targeted surveys in the middle and at the end of training to assess the success and impact of the programme (available from: https://github.com/open-life-science/cohort-surveys). OLS, Open Life Science.
(PDF)

S9 Text OLS post OLS-4 anonymous feedback, reporting, and share out form. The mentee feedback form sent to mentees at the end of the OLS-4 cohort for OLS for anonymous feedback and reporting. OLS uses targeted surveys in the middle and at the end of training to assess the success and impact of the programme (available from: https://github.com/open-life-science/cohort-surveys). OLS, Open Life Science.
(PDF)

S10 Text OLS post OLS-4 mentor survey form. The mentor feedback form sent to mentors at the end of the OLS-4 cohort for OLS. OLS uses targeted surveys in the middle and at the end of training to assess the success and impact of the programme (available from: https://github.com/open-life-science/cohort-surveys). OLS, Open Life Science.
(PDF)

S11 Text ESCALATOR EXPLORER track feedback form. The feedback form for one of the tracks of the ESCALATOR mentorship programme (EXPLORER). ESCALATOR used this feedback form to assess participation and evaluate effectiveness of this mentorship track. Similar forms will be developed for each of this programme’s tracks to assess success and impact.
(PDF)
