# Supplementary material for: Ten simple rules for establishing a mentorship programme
Source: PLoS Comput Biol. 2022 May 12;18(5):e1010015. doi: 10.1371/journal.pcbi.1010015 (PMC9098017; doi:10.1371/journal.pcbi.1010015)
Supplement: S1 Text — In order to demonstrate applications of each of the rules, for each rule, a brief case study is provided from one or more of the programmes involved in this article as examples. However, as all 4 programmes vary in some of their specifics, more in depth information about each of the programmes as they relate to each rule is provided in this appendix table. (PDF) [file pcbi.1010015.s001.pdf]

S1 Text. Programme specific information relating to each rule. In order to demonstrate applications of each of the rules, for each rule, a brief case study is provided from one or more of the programmes involved in the article as examples. However, as all four programmes vary in some of their specifics, more in depth information about each of the programmes as they relate to each rule is provided in this appendix table.

	**ESCALATOR**	**Deep Learning Indaba (DLI)**	**Open Education for a Better World (OE4BW)**	**Open Life Science (OLS)**
Rule 1: Define the programme vision and scope	ESCALATOR (https://escalator.sadilar.org) serves to address the need for an active, inclusive community of practice in Digital Humanities in South Africa. The programme organisers used various tools typically applied in programme design and monitoring and evaluation to understand the absence of a community of practice (the problem). Tools used to identify the cause included a fishbone diagram and the "Five Whys" technique. The team developed a Theory of Change to show the desired outcome (including short- and medium-term impacts) and the causal relationship between the inputs, activities, and outputs and the intended outcome and impact.<br><br>A full-day virtual meeting was designed to learn from existing mentorship programmes in various research and education fields. Invaluable insights were gained in terms of differences and similarities in scope, processes, challenges, and	The DLI mentorship programme (https://deeplearningindaba.com/mentorship) supports the long-term vision and mission of the Deep Learning Indaba organisation. The programme was specifically designed to strengthen machine learning in Africa by empowering the community. The official mission of DLI is available on the website at https://deeplearningindaba.com/about/our-mission/.	OE4BW (https://oe4bw.org) was established in 2018 with two main objectives:<br>● to support capacity building in developing, reusing, and adapting Open Educational Resources (OER); and,<br>● provide expert guidance to project leaders in developing resources with a social impact that address one or more United Nations (UN) Sustainable Development Goals (SDGs).	OLS (https://openlifesci.org) brings together mentors and mentees from different disciplines to<br>● gain essential skills and knowledge to create, lead, and sustain an Open Science project;<br>● connect with members across various projects, geography, backgrounds, and identities; and,<br>● empower each other to become Open Science ambassadors in their local communities.<br><br>In the course of 16 weeks, mentees undergo a series of training, skill-building, and personal mentorship calls while applying the learned skills in a project of their interest in real-time.

	ESCALATOR	Deep Learning Indaba (DLI)	Open Education for a Better World (OE4BW)	Open Life Science (OLS)
	opportunities.			
Rule 2: Develop the organisational structure once the desired outcomes have been defined	ESCALATOR is the only programme described in this article designed and implemented as part of a service provider agreement. Therefore, the management team consists of members from the client, the South African Centre for Digital Language Resources (SADiLaR), and the service provider, Talarify. The programme is funded as a multi-year initiative through SADiLaR, one of the South African Research Infrastructure Roadmap entities. These entities form part of a larger strategic drive by the South African Department for Science and Innovation. The ESCALATOR Management team meets once a week to discuss progress. Feedback is provided quarterly to the SADiLaR Steering Committee and the Audit, Risk and Finance committee. No additional explicit or formal structure has been established within the ESCALATOR programme, although it is envisioned that those will emerge as the programme grows. An open invitation is extended to the community to get involved in various roles.	The steering committee was established by colleagues who knew each other through previous collaboration. Steering committee members shared values and were motivated to contribute to the cause by developing a mentorship programme. The members agreed upon reasonable time contributions to get the programme off the ground and keep it running. Since membership is voluntary, in the DLI programme's experience, sustainable commitment stems from a vested interest in the programme's mission. There is no fixed term length, and steering committee members can step down at any time. Documentation is available to facilitate handovers.	During the first year of its implementation, OE4BW's organisational structure was small, consisting only of its founders. As interest in the programme grew, the organisational structure has evolved to introduce hub coordinators and the advisory board. Hub coordinators help manage processes, such as conducting regular follow-ups on projects and monitoring mentor-mentee dynamics for projects allocated to specific geographical or topical hubs by the programme organisers (https://oe4bw.org/projects). Advisory board members (https://oe4bw.org/advisory-board) provide strategic guidance to the programme organisers.	OLS was launched in 2019 under the leadership of three founders who co-developed the programme. In the first iteration, the programme onboarded 20 open science practitioners as mentors to guide 20 projects led by individuals and groups of mentees. In 2020, the leadership team applied for small-scale funding to provide microgrants for the mentees to facilitate their participation (https://zenodo.org/record/4778472). In 2021, one more member was onboarded in the leadership team to help coordinate the growing number of mentees and the mentor community. By the end of four iterations, the programme was awarded large-scale funding to hire a programme coordinator to support governance development with a steering committee that reflects a more representative membership from their community (https://zenodo.org/record/5267934).

	ESCALATOR	Deep Learning Indaba (DLI)	Open Education for a Better World (OE4BW)	Open Life Science (OLS)
Rule 3: Plan activities to support programme goals	ESCALATOR is an extensive programme encompassing a wide range of activities to support the development of a national community of practice. The mentorship programme, known as the "Digital Champions Initiative" (DCI), is the flagship activity of ESCALATOR and consists of six tracks. Although the initial project proposal only included a single mentorship track, it was adapted based on feedback from the community, a needs assessment and an investigation into the expected target audience for the mentorship programme. Each mentorship track focuses on a specific sub-group of the community, e.g. complete novices, more experienced researchers and practitioners, or those in research support roles. Activities for each track will be developed based on the needs identified for the respective sub-group to whom the track is targeted. Where possible, the ESCALATOR team will partner with existing and emerging mentorship programmes and adopt activity schedules that have already proven successful. It may be necessary to	DLI aims to strengthen the African machine learning community by developing relevant fundamental skill sets. The programme matches mentees and mentors for short-term personalised interactions across various topic areas. These may include:<br>● research study design;<br>● scientific writing;<br>● proposal development;<br>● curriculum vitae (CV) creation;<br>● research poster or presentation preparation;<br>● applications for postgraduate studies, internships, or jobs;<br>● interview coaching; or,<br>● career counselling.<br><br>The programme includes one-to-one transactional interactions for which mentees and mentors receive guidelines to support preparation. Activities can consist of watching tutorial videos, reviewing tutorials and preparing a tangible output for the mentor to review. Mentees are expected to have at least a partial draft ready to present to the mentor (e.g. CV, research proposal, presentation, etc.). The programme's guides for mentors and mentees are	As part of the application process and entry requirements, prospective mentees are referred to a range of free and open short courses on OER (https://oe4bw.org/application) to help them acquire the necessary knowledge and skills for meaningful participation in the programme. During each programme cycle, hub coordinators provide regular support to mentees and mentors, and additional capacity development activities for mentees and mentors are organised: https://oe4bw.org/webinars/ and https://oe4bw.org/event-videos/.	OLS provides training on methods and best practises in open science through online cohort calls, which feature collaborative group discussions and networking. Diverse experts from various international initiatives are invited as guest speakers to share their insights and experience related to open science topics. Mentors from the programme guide mentees, and matches are made based on specific skill sets, project needs and accessibility requirements. Assignments and worksheets offered in the program ensure that our participants systematically develop and lead collaborative and community-oriented projects and ask for specific support from mentors and experts affiliated with the programme. Materials are shared via the project website, GitHub repositories, YouTube and Zenodo. All resources are published under Creative Commons with attribution (CC-BY) or Open Source Initiative licences.

	ESCALATOR	Deep Learning Indaba (DLI)	Open Education for a Better World (OE4BW)	Open Life Science (OLS)
	expand the number of tracks based on new knowledge emerging about the community.	openly licensed and available for re-use by other initiatives. These guides can be customised as needed. The activities and supporting documents were designed through focus groups and trialled in two pilot rounds.<br><br>● The mentor preparation document; and,<br>● The mentee preparation document.		
Rule 4: Recruit mentees with success in mind	Digital Humanities and Computational Social Sciences communities in South Africa are relatively small and somewhat fragmented. Therefore, the first part of ESCALATOR focused on growing awareness and getting to know the community.<br><br>"Lite" versions for two mentorship tracks with minimal entry requirements were launched - EMPOWER and EXPLORER. They did not include the recruitment of mentees for structured pairing with mentors. Instead, they focused on awareness creation events to introduce the concept of mentorship and show the benefits of joining a	Mentee applications are screened to ensure they are part of the African machine learning, artificial intelligence, or computational neuroscience communities. Mentees' expectations must be in line with the programme's scope, and they should have at least a partial draft of the task for the mentor to review. The draft is essential to ground interactions, as sessions are very short, and it helps to provide structure. To avoid a mentee being unprepared for a session, resources are shared to help them get to a point where they will gain the most from the mentorship. Mentees can apply and reapply as many times as necessary. The DLI	OE4BW uses carefully designed application forms to assess the attributes and eligibility of potential mentees to the programme. Prospective mentees need to provide detailed information about their proposed projects, including a project plan and their motivation for joining the programme (see the application form here: https://oe4bw.org/application-form-developers/). The mentees projects are chosen based on: their expected social impact, maturity of the idea for the OER, and estimation of the project feasibility. Furthermore, the projects need to align with one of the UN SDGs.	Mentees join the OLS programme with a specific research-related (including open software, hardware, community building, documentation, events) project they are already developing or interested in getting started. The initial application prompts our applicants to indicate the status of their projects, what aspect of their research they want to develop through open research practises and what impact they want to have in their communities. In addition, they are allowed to request microgrants to buy hardware, internet access or other expenses required for enabling participation in OLS or developing their projects.

	ESCALATOR	Deep Learning Indaba (DLI)	Open Education for a Better World (OE4BW)	Open Life Science (OLS)
	community of practice. The EXPLORER registration form is available online.<br><br>The EDUCATOR track, launched in phase two of ESCALATOR, follows a more structured approach. Applicants submit proposals detailing their goals and expectations. Some questions in the application form were adopted from the OLS programme application form. To increase the odds for high quality and relevant applications, open, virtual question and answer sessions were hosted. A Frequently Asked Questions document is available online. A selection committee consisting of members from the OER and South African DH communities will evaluate applications.	programme complies with GDPR.		

	ESCALATOR	Deep Learning Indaba (DLI)	Open Education for a Better World (OE4BW)	Open Life Science (OLS)
Rule 5: Develop a mentor support strategy that goes beyond simple recruitment	Phase one of ESCALATOR's Digital Champions Initiative mentorship tracks was launched as "lite" versions (i.e. not typical one-on-one mentoring). No external mentors have been recruited, and the programme management team has taken on mentorship roles where required. As community members join activities and communication platforms (e.g. Slack), spontaneous peer mentoring has started to emerge. As the community grows and the programme matures, the management team will learn about the availability of potential mentors for mentorship tracks under development. More formal mentor support strategies will need to be developed for future tracks. The programme will be looking to research existing mentorship initiatives for guidance in this respect.	Mentors receive resources to help them prepare for sessions, and there are open channels of communication with the programme administrators to address concerns. Mentors can reject matches if they feel uncomfortable working with a specific mentee for any reason. Although not implemented in DLI yet, mentors expressed an interest in an online platform/forum where they can pose questions to each other. The suggestion came up during focus groups (in the pilot stage) and direct communication. It is not yet implemented as the central reservation is that not all mentors will engage with the platform, and questions may go unanswered. It will also take time to monitor and evaluate, and currently, it is easier to deal with mentor requests directly.<br><br>Mentors are provided with a mentor preparation document.	OE4BW hub coordinators discuss the programme's goals, objectives, roles, responsibilities, and expectations with mentors and mentees during the first round of online interactions. Hub coordinators maintain open channels of communication with mentors to address any concerns that may arise throughout the six-month programme cycle. Additional capacity development activities for mentors focus on topics such as mentoring relationships and strategies: https://youtu.be/G-lOMzD_PH8.	In delivering training and mentoring, the programme involves open science practitioners who provide real-world examples and help integrate contextualised knowledge to design and lead open research in local communities. They are onboarded as mentors, experts, and advisors in the project and provided a comprehensive overview of resources available to them. They are given training in mentorship and coaching organised with professional trainers. Graduates from each cycle are invited as mentors and provided a co-mentor, if required, to support their participation. A clear opportunity for offboarding or leaving the programme allows mentors to take a break as needed. External funding enables OLS to offer mentors an honorarium to recognise and reimburse their time and investment in the programme.<br><br>A unique feature of the OLS structure is the strong focus on community. Although mentors are assigned to specific project teams or individuals, they are part of a much larger online community. Through asynchronous interaction on the Slack workspace, they have constant access to peer

	ESCALATOR	Deep Learning Indaba (DLI)	Open Education for a Better World (OE4BW)	Open Life Science (OLS)
				mentoring and not only receive support with regards to their mentorship role within OLS, but also for their own projects, work situations, and more. Everyone who has participated in OLS can join the general channels on the Slack workspace which means everyone has access to the community with diverse skill sets and perspectives. To this effect mentorship takes place on a daily basis beyond the formal programme that runs for 16 weeks.
Rule 6: Develop and evaluate mentor-mentee matching strategies as an ongoing process	Still to be developed for the same reasons mentioned above in Rule 5.	DLI requires prospective mentors to review onboarding information. Following this, mentors complete a sign-up form to specify their expertise and availability. Mentees are required to read through preparation materials before completing an online application. Applications are open on a rolling basis throughout the year. Mentees can apply anytime as long as it is no shorter than three weeks before their deadline. The time limit ensures mentor availability. DLI reviews applications and screens available mentors to find a suitable match. If a suitable mentor is available, a mentee receives an email connecting them with the	Hub coordinators are primarily responsible for monitoring and evaluating mentee-mentor matches during regular online follow-up sessions (every 4-6 weeks). An open line of communication is maintained throughout the programme cycle. Mentors and mentees can approach hub coordinators independently or jointly to discuss any concerns that may arise.	Participants apply to the programme by expressing their expectations from a mentor in the programme. Mentors are involved in reviewing applications and have an opportunity to express interest in projects they would like to mentor. Mentors and mentees are matched based on common interest, ensuring that the skills, research topics, preferred languages and time zones are sufficiently aligned. Mentors support mentees by checking in on their progress, offering guidance, and connecting them with others in their network. Whenever required, mentors invite experts or other mentors to their meetings to ensure additional expertise is available for their mentees.

	ESCALATOR	Deep Learning Indaba (DLI)	Open Education for a Better World (OE4BW)	Open Life Science (OLS)
		mentor within one week of submitting the application form. Mentors offer one call, with an optional follow up. Beyond that, the mentor is under no obligation to support the mentee.<br><br>A feedback form is sent to each mentee following interactions with a mentor. It collects both text-based responses and scores. If a mentor is scored poorly, programme administrators are notified. An automated email is sent to the mentee to request more information and encourage reapplication. It is worth noting that the exact mentor-mentee match will not be made again. Throughout the programme, mentors and mentees are encouraged to report any misgivings.		

	ESCALATOR	Deep Learning Indaba (DLI)	Open Education for a Better World (OE4BW)	Open Life Science (OLS)
Rule 7: Consider the role that technology will play	The different mentorship tracks will cater to participants displaying varying experience levels with online collaboration and digital platforms. Technology for each track will be selected based on the track aims and according to the audience's level of expertise and needs.<br><br>For ESCALATOR as a whole, the project team primarily relies on tools for which paid subscription by participants is not necessary. In some instances, the programme takes on the responsibility to pay for platforms that enable sharing resources with community members, such as Zoom (for online meetings) and Google Workspace (for sharing collaborative documents, spreadsheets, and forms).<br><br>Where possible, materials (including talks, slides, and documents) are made available for synchronous or asynchronous offline consumption should the internet or electricity fail, or if community members are unable to participate in live events.	Most processes are automated using Google scripts. New open-source solutions that DLI and other programmes could utilise are under development. International mentors are encouraged to be mindful of the challenges most African mentees face regarding connectivity and electricity access. Mentors generally respond positively to such advice.	Decisions about technological choices require nuanced considerations since participants in the OE4BW programme come from many different countries and continents. Mentors, mentees and hub coordinators are free to select tools that work best for them for synchronous and asynchronous engagements and take existing limitations such as access to relevant infrastructure, access to the internet, cost of bandwidth and time-zone differences into account. Such tools often include Zoom and WhatsApp to support live interactions (online meetings), with emails and Google documents or spreadsheets to support asynchronous engagements and collaborations during the programme cycle.	Online training calls are delivered in English via Zoom that integrates Otter.ai for the live transcription. Live transcription is particularly crucial for people with low internet bandwidth, hearing accessibility needs, or those who don't use English as their primary language and prefer to follow audio along with the written text. A collaborative document (e.g. HackMD or Etherpad) with a clearly defined schedule is established for each call to facilitate shared note taking. Calls are recorded and shared via YouTube for those who could not attend the call in real-time or would like to re-watch all or part of the call. Self-paced learning is facilitated by pairing training sessions with assignments that help mentees reflect on lessons from the respective calls. Participants use GitHub to record their progress and engage with each others' projects. GitHub training is provided at the beginning of the course to ensure everyone is familiar with the platform and can host simple Git pages for their projects. The programme team uses a shared Google Workspace to manage and store their resources and gather feedback from all participants in a centralised location. Training materials

	ESCALATOR	Deep Learning Indaba (DLI)	Open Education for a Better World (OE4BW)	Open Life Science (OLS)
	The range of hardware and software used by the target audience of ESCALATOR can range quite dramatically. Specifications to consider include:<br>● RAM, processing power, age, battery status, and status of firmware updates of laptops, PCs, mobile devices;<br>● operating system versions (including the ability to update these based on hardware age and other criteria);<br>● audio and visual setup;<br>● access to stable and sufficiently fast internet connectivity; and,<br>● access to consistent electricity (especially when internet access or computer battery power directly depends on continued access to power).			are shared via Google Drive and cross-posted on Zenodo under an open licence. The choices of technology depend on what tools are used by each specific project, their user-friendliness and availability across different geographic locations.

	ESCALATOR	Deep Learning Indaba (DLI)	Open Education for a Better World (OE4BW)	Open Life Science (OLS)
Rule 8: Ensure communication processes are in place	The ESCALATOR programme communication strategy provides a link to the Code of Conduct, describes how community members can get in touch with the project team, lists links to project resources, and manages expectations around communication from the team. The communication manager at SADiLaR and the ESCALATOR programme manager meet bi-weekly to keep track of activities, opportunities, and communication platforms and strategies.<br><br>Marketing of events is done through the SADiLaR mailing lists, the Digital Humanities Association of South Africa, via the ESCALATOR Twitter account (@DHCSSza) and Slack workspace.<br><br>Communication processes for Digital Champions Initiative's mentorship tracks will be customised to the audience needs and experience.	Website, Twitter, and Facebook accounts are regularly updated with information and opportunities.	Website, mailing lists, Facebook and Twitter accounts, and a WhatsApp group are used by the programme management team to communicate and discuss or share relevant programme activities and developments. Mentors and mentees are free to decide which technological tools to use that effectively support synchronous and asynchronous operation modes.	The OLS website is developed on GitHub and hosted at https://openlifesci.org . The website is used for open and transparent communication of different roles and opportunities in the programme. The OpenReview platform is used for receiving and reviewing applications, and maintaining communication with the applicants. A community participation guideline, code of conduct, and list of responsibilities for all our participants are communicated clearly via the website and shared via emails. A team email is shared in all relevant resources to allow participants to contact the programme team. Twitter and a public mailing list are used to announce new calls for applications and to share resources online. At the beginning of each week, the programme team shares updates and general information via a cohort-specific mailing list. Customised and in-person support is provided by maintaining communication via Slack channels, and personal mentorship calls for check-ins are hosted using the online platform of the mentees' choice. A shared calendar communicates programme schedules across different time zones. Training

	ESCALATOR	Deep Learning Indaba (DLI)	Open Education for a Better World (OE4BW)	Open Life Science (OLS)
				calls are hosted via Zoom.
Rule 9: Design a monitoring and evaluation plan	The management team performs a quarterly review of the programme. The report is shared with the SADiLaR Steering, and the Audit, Risk and Finance committees for internal discussion and feedback. A more formal midterm review was executed at the end of December 2021 through a series of meetings to consider the programme's status and the impact of COVID-19 on planned activities. The outcome of these strategic meetings informed a revision of the project plan, Theory of Change, Logical Framework, and reprioritisation of activities for 2022 and beyond. The report is currently under development for sharing with the broader community and will be published online when it is available.<br><br>ESCALATOR was designed with agility in mind, partly since it commenced at the outset of the COVID-19 pandemic and partly because the community is young and the programme management team realised the project plan was based on certain assumptions about the optimal way to achieve the desired impact. As the community grows and	Based on feedback from focus groups and lessons learned, the programme has evolved since the first two pilots. The steering committee convenes a needs assessment basis to assess the process and suggest and implement any changes. Feedback forms from mentors and mentees are continuously reviewed to ensure the programme is as accessible as possible and to monitor the quality of interactions.	The first two programme iterations (2018 and 2019) were evaluated through feedback surveys for mentors and mentees. Following this evaluation process, several changes were made to the programme, including the introduction of hub coordinators to manage the programme scale. Hub coordinators provide regular feedback about the projects in different hubs to the programme organisers, and their feedback is also discussed during the advisory board meetings. Mentors and mentees have an opportunity to provide overall impressions about the programme during the final OE4BW event that usually takes place as an in-person meeting (2018-2019) or online (2020-2021).	The success and impact of OLS are assessed using targeted surveys for mentees and mentors in the middle and end of their training. Online interactions and engagements are supported via Slack channels, and mentors keep track of mentees' progress through regular check-ins. A dedicated channel to receiving anonymous reporting and feedback is monitored to provide additional support to our participants. These activities are summarised and published in annual reports. Feedback from the participants is also considered and integrated when the programme's next iteration is planned. A research project has been designed to conduct a long-term impact study to specifically report on training and mentoring practises in open science that are successful and transformative for local communities.

	ESCALATOR	Deep Learning Indaba (DLI)	Open Education for a Better World (OE4BW)	Open Life Science (OLS)
	becomes more active in the programme, more diverse input becomes available to help shape activities and outputs. Community members are constantly invited to provide feedback or participate in the planning of events.<br><br>Formal (optional) feedback forms are available for the EXPLORER and EMPOWER tracks.			
Rule 10: Think about funding and long-term sustainability	The ESCALATOR programme is funded until 2023 by the South African Centre for Digital Language Resources (SADiLaR) - one of the national government's Research Infrastructures. The programme management team is considering various options to extend the programme as sustainable communities take many years to develop.	Most of the processes are automated, including emails, selection tools, reminders and feedback forms. Automation alleviates the time commitment of the steering committee and thus promotes long-term sustainability as the time commitment is minimal in the long run. It took time to develop these strategies, and hence an extensive pilot programme is encouraged to iterate many times over the processes to streamline and find potential for automation.	Since its inception in 2018, the founders, advisory board members, hub coordinators, and mentors have sustained OE4BW's activities through their voluntary contributions (time and expertise) to the programme. Before 2020, the programme founders secured a small amount of external funding to help subsidise in-person participation for selected project mentees and mentors in the final OE4BW event in Vipava, Slovenia (https://unesco.ijs.si/event/open-education-design).	In the initial phase in 2019, OLS ran with 100% volunteer labour and minimal infrastructure support. By successfully piloting the program and running a cohort with 20 international projects led by 29 members from five continents, OLS highlighted demand for structured training and mentoring in open science. The programme team demonstrated the project's viability, assessed the impact, and openly published an annual report to share lessons learned with the community. The subsequent phase focused on maintaining the quality of activities and acquiring funding through independent funding schemes. The grant allowed the programme to support mentees' participation and offer honoraria to mentors to recompense for their time. During this

	ESCALATOR	Deep Learning Indaba (DLI)	Open Education for a Better World (OE4BW)	Open Life Science (OLS)
				time, collaborations were developed with international institutions and local communities, and support was pursued from universities and funding bodies to make the programme more sustainable over the long term. In the third (current) phase, the project team secured extensive funding to employ team members, support the programme's sustainability and conduct research to record the impact of training and mentoring in open science.
