# Supplementary material for: Ten simple rules for establishing a mentorship programme
Source: PLoS Comput Biol. 2022 May 12;18(5):e1010015. doi: 10.1371/journal.pcbi.1010015 (PMC9098017; doi:10.1371/journal.pcbi.1010015)
Supplement: S2 Text — OE4BW uses carefully designed application forms to assess the attributes and eligibility of potential mentees to the programme. Prospective mentees need to provide detailed information about their proposed projects, including a project plan, and their motivation for joining the programme. The mentees’ open education resources projects are chosen based on their social impact, maturity of the idea for the course, and estimation of the project feasibility. Furthermore, the projects need to align with one of the UN SDGs—part of the programme’s vision and scope. OE4BW, Open Education for a Better World; SDG, Sustainable Development Goal; UN, United Nations. (PDF) [file pcbi.1010015.s002.pdf]

APPLICATION FORM

for developers

University of Nova Gorica (UNG) and UNESCO Chair on Open Technologies for OER and Open Learning at the Jožef Stefan Institute (JSI) are announcing a new round of the Open Education for a Better World international online mentoring program. The program supports the development and implementation of freely accessible modules and resources for online education on topics with social impact according to the UN Sustainable Development Goals (SDGs). The program will take place from January to the end of June 2022.

The OER projects will be chosen based on: their social impact, maturity of the idea for the course, estimation of the project feasibility.

Candidates for developers are invited to **apply by December 13, 2021**.

The whole program is based on voluntary work. We are not able to provide any financial compensation for your participation. If you have any questions or concerns please don't hesitate to contact Anja Polajnar or Ana Fabjan.

"*" indicates required fields

PART A: PERSONAL INFORMATION

Name *

Prefix

First

We use cookies to ensure that we give you the best experience on our website.

OK

Gender *

◯ Male

◯ Female

Residence *

City

Country

Email *

Please provide.

Phone *

Please provide.

In which language(s) are you fluent? *

Education *

◯ Vocational

◯ BSc

◯ MSc

We use cookies to ensure that we give you the best experience on our website.

OK

I apply as *

- ○ a student
- ○ an employee
- ○ an organisation
- ○ other

Have you already participated in the OE4BW program as a developer? *

- ○ Yes
- ○ No

Your experience *

- ○ Beginner - no experience with OER development
- ○ Intermediate - already have experience with OER, but have not yet developed it
- ○ Advanced - have already developed a OER, but would like to improve it

Participation *

Briefly describe how you think participation in OE4BW will help you achieve your goal.

In the future I would like to gain formally recognized qualifications related to knowledge of Open Education and OER (more than one answer possible) *

- ☐ I am interested in pursuing a Master's degree that focuses on these topics.
- ☐ I am interested in completing a certified shorter form of training on these topics (specialization).
- ☐ I am interested in earning badges.
- ☐ I am only interested in knowledge and skills without the need for certification.

PART B: OER PROJECT

Title of your OER project *

Title of the open course or open resource that you would like to develop.

We use cookies to ensure that we give you the best experience on our website.

OK

Target audience *

Briefly describe the audience for your course or resource (educators, students, administrators, policy makers, researchers, etc.). The content could be helpful to many groups, but try to be as specific as possible. Who do you think might be the main beneficiary?

Motivation *

Briefly describe your motivation for creating this course or resource. What need, opportunity, or challenge do you want your OER project to address?

Summary *

Description of your project.

0 of 2800 max characters

Keywords *

Name at least 5 keywords describing the contents of your project.

We use cookies to ensure that we give you the best experience on our website.

OK

Project status *

- ◯ Idea
- ◯ Idea + structure of the course or resource
- ◯ Idea + structure + materials
- ◯ Course or resource already partially developed

Project type *

Please indicate how many people will develop the OER project.

- ◯ Individual
- ◯ Team

Development Timeline *

Imagine a six-month development plan (from January to June) and think about what you can accomplish in that time frame. Please specify what activities are required for your OER project, what help you will need to accomplish them, and how long each activity will take. Your main goal is to develop something concrete by the end. The plan can be very general and it can be changed. The goal is that you, the developer, and your mentor(s) to have a shared understanding of what you are trying to do.

0 of 2800 max characters

Connection to SDGs *

We use cookies to ensure that we give you the best experience on our website.

OK

- SDG 1: No Poverty
- SDG 2: Zero Hunger
- SDG 3: Good Health and Well-being
- SDG 4: Quality Education
- SDG 5: Gender Equality
- SDG 6: Clean Water and Sanitation
- SDG 7: Affordable and Clean Energy
- SDG 8: Decent Work and Economic Growth
- SDG 9: Industry, Innovation and Infrastructure
- SDG 10: Reduced Inequality
- SDG 11: Sustainable Cities and Communities
- SDG 12: Responsible Consumption and Production
- SDG 13: Climate Action
- SDG 14: Life Below Water
- SDG 15: Life on Land
- SDG 16: Peace and Justice Strong Institutions
- SDG 17: Partnerships to achieve the Goal

PART C: CONSENT AND SUBMISSION

Consent *

I will attend scheduled meetings with the mentor(s) and hub coordinator(s) and I will implement the OER project by the end of June 2022, which will be openly available under a Creative Commons license.

- I agree

Confirmation *

The whole program is based on voluntary work and there will be no financial compensation for developing and implementing OER.

- I understand

Course completion certificate *

Please upload the proof of completing one of the listed courses on the OE4BW website in

We use cookies to ensure that we give you the best experience on our website.

OK

Max. file size: 5 MB.

Upload your CV *

Please upload your CV.

Choose file No file chosen

Max. file size: 5 MB.

SUBMIT

We use cookies to ensure that we give you the best experience on our website.

OK
