# Supplementary material for: Ten simple rules for establishing a mentorship programme
Source: PLoS Comput Biol. 2022 May 12;18(5):e1010015. doi: 10.1371/journal.pcbi.1010015 (PMC9098017; doi:10.1371/journal.pcbi.1010015)
Supplement: S3 Text — The registration form for one of the tracks of the ESCALATOR mentorship programme (EXPLORER). This track had no minimum requirements for entry, and a selection process was not implemented. The form was used to assess participation only. (PDF) [file pcbi.1010015.s003.pdf]

EXPLORER Registration Form

Last updated: January 2022

Description: This is the registration form for participants in the ESCALATOR Digital Champions Initiative EXPLORER track. For more information please visit our website - https://escalator.sadilar.org/champions/explorer/.

* Required

Registration and feedback for the EXPLORER track is optional, but by letting us know that you are working through the materials, we are able to support you better. By providing feedback you can help us to improve the programme and have a bigger impact on our community.

Thank you for letting us know that you're interested in the ESCALATOR Explorer Track.

We hope you will be following us on Twitter - https://twitter.com/DHCSSza.

You can also join our Slack Workspace - https://escalator.sadilar.org/post/connect-with-the-community/

1. *Email: ___________________
2. First name: ___________________
3. Surname: ___________________
4. *How FAMILIAR are you with topics covered in the ESCALATOR Explorer track? (*❒ Not at all familiar; ❒ Slightly familiar; ❒ Moderately familiar; ❒ Very familiar; ❒ Extremely familiar*)
   - ☐ Digital Humanities
   - ☐ Computational Social Sciences
   - ☐ Computational Thinking
   - ☐ Digital Scholarship
   - ☐ Open Access
   - ☐ Open Research
   - ☐ Open Educational Resources
   - ☐ Research Data Management
   - ☐ Reproducible Research
   - ☐ Digital Humanities/Computational Social Sciences in Africa

5. *How INTERESTED are you with topics covered in the ESCALATOR Explorer track? *(❒ Not at all interested; ❒ Slightly interested; ❒ Moderately interested; ❒ Very interested; ❒ Extremely interested)*
    - ☐ Digital Humanities
    - ☐ Computational Social Sciences
    - ☐ Computational Thinking
    - ☐ Digital Scholarship
    - ☐ Open Access
    - ☐ Open Research
    - ☐ Open Educational Resources
    - ☐ Research Data Management
    - ☐ Reproducible Research
    - ☐ Digital Humanities/Computational Social Sciences in Africa

6. Do you have any questions about the programme?

    ____

    ____

7. *Would you like to attend an informal meeting to meet some of the ESCALATOR team members and ask questions about the EXPLORER track or anything else related to digital scholarship in Humanities or Social Sciences?
    - ☐ Yes
    - ☐ No
    - ☐ Maybe

8. *By completing this form you agree that the ESCALATOR team can contact you about the ESCALATOR programme and the Explorer track. *(We will be sharing information about upcoming meetings and opportunities related to the programme. You can unsubscribe from receiving further information at any time.)*
    - ☐ Yes
    - ☐ No
