# Supplementary material for: Ten simple rules for establishing a mentorship programme
Source: PLoS Comput Biol. 2022 May 12;18(5):e1010015. doi: 10.1371/journal.pcbi.1010015 (PMC9098017; doi:10.1371/journal.pcbi.1010015)
Supplement: S4 Text — The application form for one of the tracks of the ESCALATOR mentorship programme (EDUCATOR) for 2022–2023. ESCALATOR implemented a more structured approach for this track as opposed to previous tracks, and mentees have to submit proposals detailing what they would like to achieve by joining the mentorship programme and what they expect from the programme team. Some of the questions in the application form were adopted from the OLS programme application form. Selection of mentees will be done by the programme team. (PDF) [file pcbi.1010015.s004.pdf]

DH OER Champions application form 2022/2023

Last modified: January 2022

Website: https://escalator.sadilar.org/champions/dh-oer/
Application form: https://forms.gle/iWXe5y8RfP9a1RVe7
Note: *Some questions on this form was adapted from the Open Life Science programme's application form available at https://github.com/open-life-science/application-forms*

Instructions

- **Please feel free to make a copy of this document to facilitate collaborative proposal development with team members. Answers can be copied into the application form available at https://forms.gle/iWXe5y8RfP9a1RVe7**
- **Only application forms submitted via the form at https://forms.gle/iWXe5y8RfP9a1RVe7 will be considered**
- **Example answers in green for clarification**

1. Name and surname (or all the names and surnames in the case of a group application) *

   e.g. Jako Olivier (lead); Anelda van der Walt

2. E-mail address (or all the e-mail addresses in the case of a group application) *

   e.g. Jako.Olivier@nwu.ac.za (lead); anelda@talarify.co.za

3. Institution and faculty/department of each applicant (or organisation if not a university) *
   Please indicate the affiliation of the team lead explicitly

   e.g. North-West University (lead); Talarify

4. Project/OER title *

5. List 3 keywords (or key phrases) related to the OER that you will create. Please separate with semicolons (;)

   e.g Natural language processing; isiXhosa; Python

6. Why do you want to create a DH OER? Provide a motivation of maximum 300 words. *

7. What problem(s) are you solving with this project? Please describe in a maximum of 200 words. *

8. What is the current status of your project or work? Choose one option *
   a. I/we have a great idea
   b. I/we am working towards a first OER
   c. I/we just launched an OER
   d. I/we are working on a new release/feature of an existing OER
   e. I/we are maintaining work on an existing OER

9. What are your learning goals for the OER? *

10. What do you expect from this programme? *

11. Have you been involved in developing or using OER before? Please explain.

    Applicants with no prior experience with OERs will not be penalised. This question is merely asked to inform our planning.

12. Description of what you are planning (maximum 600 words). *

13. Any other comments to the evaluation panel

14. Have you been involved in any activities offered through the ESCALATOR programme?
    a. Yes
    b. No
    c. I don't know what the ESCALATOR programme is

15. How/Where did you hear about the DH OER Fellowship?

16. My/our line manager(s) or supervisor/promoter supports my participation in the DH OER Fellowship
    a. Yes, I have discussed my application with them and they support it
    b. No, I have discussed my application with them and they do not support it
    c. I have not discussed my application with them
    d. Other:

17. I/we commit myself/ourselves to: (1) following the training as set out for this fellowship, (2) completing a DH OER, (3) conducting research and (4) making the DH OER available under a Creative Commons License. *
    a. I commit myself/We commit ourselves
