# Supplementary material for: Ten simple rules for establishing a mentorship programme
Source: PLoS Comput Biol. 2022 May 12;18(5):e1010015. doi: 10.1371/journal.pcbi.1010015 (PMC9098017; doi:10.1371/journal.pcbi.1010015)
Supplement: S5 Text — The OE4BW follow-up questionnaire/feedback survey for mentors. The first 2 iterations of OE4BW (2018 and 2019) were evaluated through feedback surveys for mentors and mentees. Following this evaluation process, several changes were made to the programme, including the introduction of hub coordinators to manage the programme scale. Evaluations using feedback surveys for mentors and mentees will also be conducted with the 2020 and 2021 cohorts. OE4BW, Open Education for a Better World. (PDF) [file pcbi.1010015.s005.pdf]

OE4BW FOLLOW UP QUESTIONNAIRE FOR MENTORS

1. On a scale of 1-10, how satisfied were you with your participation in the OE4BW mentoring program? (Optional: Comments ____)

2. On a scale of 1-10, how satisfied were you with the choice of your mentee? (Optional: Comments ____)

3. How would you describe the communication with your mentee? (choose 1 answer)

    a. We communicated regularly, with a reasonable frequency.
    b. The mentee wanted us to communicate very often. I think he/she should be more independent.
    c. The mentee didn't contact me as much as expected. I would like him/her to be more proactive.
    d. Other (please specify): _________________________

4. How would you describe the cooperation with your hub coordinator? (choose as many answers as you want)

    a. We communicated regularly, with a reasonable frequency.
    b. The mentee wanted us to communicate very often. I think he/she should be more independent.
    c. I think there was a lot of communication with hub coordinator and might be possible to reduce it in the future.
    d. Other (please specify): _________________________

5. How would you describe the progress of your mentee?

    a. His/her OER project in the OE4BW developed well and raised a reasonable level of maturity

**Strongly agree**	**Agree**	**Neutral**	**Disagree**	**Strongly disagree**

    b. His/her technical knowledge increased

**Strongly agree**	**Agree**	**Neutral**	**Disagree**	**Strongly disagree**

c. After the program he/she should be more capable of implementing new OER in the future

Strongly agree	Agree	Neutral	Disagree	Strongly disagree

d. Other comments (optional): ____

6. How would you describe connections established during the program? (choose as many answers as relevant)

    a. I believe I will stay in contact with my mentee, we might continue with the project or cooperate in another way.

    b. I would like to stay in contact with my hub coordinator and OE4BW organizers, we might establish new ways of cooperation.

    c. I would like to be connected to the whole OE4BW community to exchange information and ideas about potential cooperation.

    d. I don't have any opinion on this, I will seek contacts if needed in the future.

    e. Other comments (optional)_________________________________

7. How would you describe your experience with OE4BW in more detail?

    a. It took more of my time than expected.

Strongly agree	Agree	Neutral	Disagree	Strongly disagree

b. It was rewarding to see the progress of the mentee.

Strongly agree	Agree	Neutral	Disagree	Strongly disagree

c. It was rewarding to help to a project for social benefit.

Strongly agree	Agree	Neutral	Disagree	Strongly disagree

8. Will you like to participate in OE4BW 2020?

    a. Yes, I would like to be a mentor.

    b. Yes, I would like to contribute as a hub coordinator for projects from certain region or a certain topic.

    c. No, I don't believe I will participate.

    d. I don't know yet.

9. If you will not participate in OE4BW next year, what is the reason?

    a. I would like to, but can not due to time constraints or other personal reasons.
    b. I was disappointed last year.
    c. Other (please specify): ______________________________

10. Would you recommend participation to your colleagues or friends?

    a. Yes, I actually did.
    b. Yes, I would.
    c. No.

11. Given your experience, how would you improve the OE4BW mentoring program?

    ____

12. How would you describe the existing opportunities to learn about Open Education?

    a. There are enough opportunities.

**Strongly agree**	**Agree**	**Neutral**	**Disagree**	**Strongly disagree**

    b. I think that open on-line courses are sufficient to get this knowledge.

**Strongly agree**	**Agree**	**Neutral**	**Disagree**	**Strongly disagree**

    c. I think that shorter certified courses are needed.

**Strongly agree**	**Agree**	**Neutral**	**Disagree**	**Strongly disagree**

    d. I think a Master’s program is needed.

**Strongly agree**	**Agree**	**Neutral**	**Disagree**	**Strongly disagree**

13. In the field of open education, I would like to get more knowledge about (choose as many answers as you want)

   a. Open education strategies and policies
   b. Effective didactical practices in open education
   c. Technologies for open education
   d. Business and organizational models of open education
   e. Production of educational materials
   f. Open education in industry and business (related to Human Resource Management)
   g. Other (please specify): ______________________________________
