# Supplementary material for: Ten simple rules for establishing a mentorship programme
Source: PLoS Comput Biol. 2022 May 12;18(5):e1010015. doi: 10.1371/journal.pcbi.1010015 (PMC9098017; doi:10.1371/journal.pcbi.1010015)
Supplement: S6 Text — The OE4BW follow-up questionnaire/feedback survey for mentees. The first 2 iterations of OE4BW (2018 and 2019) were evaluated through feedback surveys for mentors and mentees. Following this evaluation process, several changes were made to the programme, including the introduction of hub coordinators to manage the programme scale. Evaluations using feedback surveys for mentors and mentees will also be conducted with the 2020 and 2021 cohorts. OE4BW, Open Education for a Better World. (PDF) [file pcbi.1010015.s006.pdf]

OE4BW FOLLOW UP QUESTIONNAIRE FOR DEVELOPERS

1. On a scale of 1-10, how satisfied were you with your participation in the OE4BW mentoring program? (Optional: Comments ____)
2. On a scale of 1-10, how satisfied were you with the choice of your mentors and the help you received from them? (Optional: Comments ____)

3. Did you achieve what you expected?

    a. I implemented my idea for OER as planned
    b. I partially implemented my idea for OER
    c. I did not achieve what I expected

4. What happened with the results of your project?
    a. Materials have been released as OER and are being used.
    b. Materials are ready to be used.
    c. Materials are not ready to be used, but development continues
    d. Materials are not ready, but further development is planned.
    e. Materials are not ready to be used and I don’t plan to continue

5. What did you benefit from OE4BW program?
    a. My technical knowledge increased
    b. After the program I feel more capable of implementing new OER in the future

**Strongly agree**	**Agree**	**Neutral**	**Disagree**	**Strongly disagree**

6. How would you describe the communication with your mentor? (choose 1 answer)

    a. We communicated regularly, with a reasonable frequency.
    b. I would like us to communicate more.
    c. I think there was a lot of communication with the mentor and might be possible to reduce in the future
    d. Other (please specify): _________________________

7. How would you describe communication with your hub coordinator?

    a. We communicated regularly, with a reasonable frequency.
    b. I would like us to communicate more.
    c. I think there was a lot of communication with the mentor and might be possible to reduce in the future
    d. Other (please specify): ________________________

8. How would you describe connections established during the program (choose as many answers as relevant)
    a. I believe that I will stay in contact with my mentor, we might continue with the project or cooperate in another way.
    b. I would like to stay in contact with my hub coordinator and OE4BW organizers, we might establish new ways of cooperation.
    c. I would like to be connected with the whole OE4BW community to exchange information and ideas about potential cooperation.
    d. I don't have any opinion on this, I will seek contact if needed in the future.
    e. Other:_______________________

9. What are your expectations regarding what you have learned in the OE4BW program?
    a. I will use what I have learned in my everyday work.
    b. I will use what I have learned from time to time.
    c. I don't see direct application of what I have learned at the moment, but it might be relevant in the future.
    d. I see no potential of using what I have learned.

10. Would you like to participate in OE4BW 2020?
    a. Yes, I would like to continue as a developer in this program.
    b. Yes, I would like to continue as a mentor.
    c. Yes, I would like to contribute as a hub coordinator for project from a certain region or a certain topic.
    d. No, I don’t believe I will participate.
    e. I don’t know yet.

11. If you will not apply / have not applied for OE4BW 2020, what is the reason?
    a. I would like to, but can not due to time constraints or other personal reasons.
    b. I was disappointed last year.
    c. Other (please specify): ______________________________

12. Would you recommend participation to your colleagues or friends?
    a. Yes, I actually did.
    b. Yes, I would.
    c. No.

13. Given your experience, how would you improve the OE4BW mentoring program?

____

14. How would you describe the existing opportunities to learn about Open Education?

    a. Yes, I would like to get a Master's degree in Open Education.
    b. Yes, I'm interested in shorter, but certified life-long-learning courses.
    c. No, I already have formally recognized qualifications in open education.
    d. No, I only want to get knowledge, but I don't need certificates.
    e. No, it is not relevant for me.

15. In the field of open education, I would like to get more knowledge about (choose as many answers as you want)
    a. Open education strategies and policies
    b. Effective didactical practices in open education
    c. Technologies for open education
    d. Business and organizational models of open education
    e. Production of educational materials
    f. Open education in industry and business (related to Human Resource Management)
    g. Other (please specify): _______________________________________
