# Supplementary material for: Ten simple rules for establishing a mentorship programme
Source: PLoS Comput Biol. 2022 May 12;18(5):e1010015. doi: 10.1371/journal.pcbi.1010015 (PMC9098017; doi:10.1371/journal.pcbi.1010015)
Supplement: S7 Text — The DLI feedback form for mentees. As part of a mentor–mentee matching strategy, DLI sends a feedback form to all mentees following interactions with the mentors. DLI continuously reviews the forms to ensure the programme is as accessible as possible and that the mentor–mentee interactions are productive. DLI management meets bimonthly to assess the programme processes and to suggest and action any changes. DLI, Deep Learning Indaba. (PDF) [file pcbi.1010015.s007.pdf]

Deep Learning Indaba Mentorship Programme Feedback Form

Please complete this feedback form after your session (including any follow-ups) with your mentor. Your feedback will help us to ensure the DLI Mentorship Programme continues to meet the needs of our large and diverse community. Your (anonymised) feedback may also be shared with audiences outside of the Indaba organisation when promoting the Programme.

This form should take 5-10 minutes to complete. If you have any questions/comments, please email mentorship@deeplearningindaba.com.

- Full name *[short answer text, required*]*
- Email address *[short answer text, required]*
- Full name of mentor *[short answer text, required]*
- Please rate the overall experience of your session: [1] dreadful, and [10] exceeded my expectations *[rating scale, required]*
    - If you rated 6 or lower, please explain why *[long answer text, optional**]*
- Were your expectations for the session met? *[options: yes / no, required]*
    - If you responded no, please describe what your expectations were going into the session and why they were not met *[long answer text, optional]*
- Describe any challenges you experienced during your session.? These challenges could have come up before/during/after the session, and could include, but are not limited to, connectivity issues, scheduling conflicts and/or language barriers *[long answer text, optional]*
- Are there ways we can improve the experience for future mentors/mentees? If yes, please describe *[long answer text, optional]*
- Do you have any other comments, concerns or queries? *[long answer text, optional]*

* required: the form cannot be submitted if left blank
**optional: the form can be submitted without completing the respective section
