# Supplementary material for: Ten simple rules for establishing a mentorship programme
Source: PLoS Comput Biol. 2022 May 12;18(5):e1010015. doi: 10.1371/journal.pcbi.1010015 (PMC9098017; doi:10.1371/journal.pcbi.1010015)
Supplement: S8 Text — The mentee feedback form sent to mentees at the end of the OLS-4 cohort for OLS. OLS uses targeted surveys in the middle and at the end of training to assess the success and impact of the programme (available from: https://github.com/open-life-science/cohort-surveys). OLS, Open Life Science. (PDF) [file pcbi.1010015.s008.pdf]

Post OLS-4 Survey for the Mentees

This is the post-cohort survey for Open Life Science cohort 4 (OLS-4). Completing this survey is mandatory to receive your program completion certificate. Your responses will be kept confidential. Only the organizers (Yo, Berenice, Malvika and Emmy) will have access to the survey results.

Email *

GDPR statement: The information provided in this form will be shared only with the OLS organisers to help them assess the impact of our program. All the information collected will be used towards improving our program for our participants. A summary will be shared in a blog post by removing any identifiable information. You can edit your response until 28 Feb 2022. Please select "I understand and I accept" before proceeding.

☐ I understand and I accept

How was your overall project leadership experience in OLS-4? *

☐ I was NOT able to work on my project idea
☐ I was able work on my project but only PARTIALLY I was able to meet MOST of my goals
☐ I was able to meet ALL my goals
☐ Other:

If you struggled to participate in OLS-4 for any reason, would you prefer to defer your graduation and participate in OLS-5 as well?

We're asking this to get an idea of numbers since we noted a little drop-off in OLS-4. We're not 100% sure why but thought it might be due to pandemic-related fatigue, burnout, or illness, so we're exploring what options we can offer. Any info you can provide would be handy, and if you answer "yes" we'll be in further contact to see how we can help out.

☐ Yes, I'd love it if there was an option to participate in OLS-5 as a mentee
☐ Not applicable - I would still prefer to graduate during OLS-4
☐ Other:

If your project plans deviated from your initial goals, please share what influenced those changes and if it was useful.

How was your overall experience with the mentor-mentee calls (this does not include cohort calls)? *

☐ Mentoring calls were not useful
☐ Mentoring calls were somewhat useful
☐ Mentoring calls were mostly useful
☐ Mentoring calls were always useful

Please share one aspect of your mentoring experience that impacted your project or you as an Open Science leader?

How was your overall experience with the cohort calls (either when you attended those or watched the recordings on YouTube)? *

- ☐ They were not useful for me
- ☐ They were somewhat useful
- ☐ They were mostly useful
- ☐ They were always useful

Which of the following topics introduced in these cohort calls were useful for your open science journey? (multiple options can be chosen) *

- ☐ Tooling and Roadmapping (open canvas, project vision etc.)
- ☐ Licensing and Code of Conduct
- ☐ GitHub and README files
- ☐ Project development: Agile and iterative project management methods & Open Aspects
- ☐ Knowledge Dissemination: Preprints, Training and Code Publishing Data management plans, software citation
- ☐ Citizen Science
- ☐ Diversity & Inclusion
- ☐ Mountain of engagement and Community interactions
- ☐ Persona and pathways and inviting contributions Mental health, self care, personal ecology
- ☐ Ally skills
- ☐ Open Leadership: Career Guidance call
- ☐ Open office/co-working hours and social calls
- ☐ Final presentation rehearsals
- ☐ Final presentation call (open and live streamed)
- ☐ Other:

Can you think of a topic that we discussed in the cohort call that is not useful for Open Science projects and leaders?

If you are happy for us to you publish your project's speed blog on our website at https://openlifesci.org/posts (as per this template: https://hackmd.io/@ols-2/speedblog-guide), please provide a link to your speed blog.

Would you be interested in joining the OLS-5 as a mentor or expert of the next cohort? *

- ☐ Yes I'd like to return as a mentor
- ☐ Yes I'd like to return as an expert
- ☐ Yes I'd like to return as a call facilitator
- ☐ Yes I'd like to return as a collaborator to run an OLS cohort for my network I am not sure yet, but ask me later
- ☐ No, I would not be able to return to OLS-5
- ☐ Other:

Would you like to be added to the OLS-alumni email group?

It's very low traffic, probably no more than 5 emails a year. - [ ] Yes - [ ] No

Would you recommend this program to others? *

- ☐ Yes
- ☐ Maybe
- ☐ No

We will send you a certificate of completion via email. Please provide your name as you wish to be printed on your certificate. *

Please provide the title of your project as you wish to be printed on your certificate. *

Anything else you would like to share with us? (we have also created an anonymous survey: [LINK TO ANON FORM] and encourage you to share any feedback, reflections and incident from the OLS-4 program that the OLS team should know.)
