# Supplementary material for: Ten simple rules for establishing a mentorship programme
Source: PLoS Comput Biol. 2022 May 12;18(5):e1010015. doi: 10.1371/journal.pcbi.1010015 (PMC9098017; doi:10.1371/journal.pcbi.1010015)
Supplement: S9 Text — The mentee feedback form sent to mentees at the end of the OLS-4 cohort for OLS for anonymous feedback and reporting. OLS uses targeted surveys in the middle and at the end of training to assess the success and impact of the programme (available from: https://github.com/open-life-science/cohort-surveys). OLS, Open Life Science. (PDF) [file pcbi.1010015.s009.pdf]

Post-OLS-4 Anonymous feedback, reporting and share out

This survey is for anonymous feedback, reporting or share out from the Open Life Science cohort 4 (OLS-4). Adding your response is optional but wholeheartedly welcome. Please take this opportunity to also report any incident that might have made you uncomfortable in the past any time during the OLS-4 program. Your responses will be kept confidential within the OLS team members (Yo, Berenice, Malvika and Emmy) and will not be shared with anyone unless requested by the respondent.

Your feedback that you would like to share with the OLS team anonymously?

Any reflection from the program that you would like to share with the OLS team that they should consider for the next cohort?

Is there any incident from OLS-4 you would like to report that might have made you uncomfortable or excluded from the program?

Anything else you would like to share with the OLS team?
