# Supplementary material for: Ten simple rules for establishing a mentorship programme
Source: PLoS Comput Biol. 2022 May 12;18(5):e1010015. doi: 10.1371/journal.pcbi.1010015 (PMC9098017; doi:10.1371/journal.pcbi.1010015)
Supplement: S10 Text — The mentor feedback form sent to mentors at the end of the OLS-4 cohort for OLS. OLS uses targeted surveys in the middle and at the end of training to assess the success and impact of the programme (available from: https://github.com/open-life-science/cohort-surveys). OLS, Open Life Science. (PDF) [file pcbi.1010015.s010.pdf]

Post OLS-4 Survey for the Mentors

This is the post-cohort survey for Open Life Science cohort 4 (OLS-4). If you have mentored more than one project, you should send separate responses for each of them. Your responses will be kept confidential. Only the organizers (Yo, Berenice, Malvika and Emmy) will have access to the survey results.

GDPR statement:

The information provided in this form will be shared only with the OLS organisers to help them assess the impact of our program. All the information collected will be used towards improving our program for our participants. A summary will be shared in a blog post by removing any identifiable information. You can edit your response until 10 days after you have submitted your response. Please select "I understand and I accept" before proceeding. *

- ☐ I understand and I accept

Email address

Name

Please provide the name of your mentee(s)

Please provide the project name of your mentees

(See here for details: https://openlifesci.org/ols-4/projects-participants/). *Sorry for asking two questions about this! It makes it much easier to correctly match up results when we invite mentees back as mentors*

How were your overall mentorship training and support experience in OLS-4? (multiple checks are possible)

- ☐ I felt supported as a mentor and the training offered in the OLS-4 was adequate
- ☐ I felt supported as a mentor but the training offered in the OLS-4 can be improved I enjoyed my participation and did not find the experience overwhelming
- ☐ I found the mentorship responsibilities overwhelming
- ☐ I did not feel supported as a mentor
- ☐ Other:

What expectations from the OLS-4 program were met and where can we do better?

How was your overall experience with the mentoring calls with your mentee? *

- ☐ Mentoring calls were not structured or constructive
- ☐ Mentoring calls were somewhat constructive
- ☐ Mentoring calls were mostly constructive
- ☐ Mentoring calls were always constructive

If you followed the cohort calls (notes or videos), Which of the following topics introduced in these cohort calls were useful for you? (multiple options can be chosen)

- ☐ Tooling and Roadmapping (open canvas, project vision etc.)
- ☐ Licensing and Code of Conduct
- ☐ GitHub and README files
- ☐ Project development: Agile and iterative project management methods & Open Aspects
- ☐ Knowledge Dissemination: Preprints, Training and Code Publishing Data management plans, software citation
- ☐ Citizen Science
- ☐ Diversity and Inclusion
- ☐ Mountain of engagement and Community interactions Persona and pathways and inviting contributions Mental health, self care, personal ecology
- ☐ Ally skills
- ☐ Open Leadership: Career Guidance call
- ☐ Open office/co-working hours and social calls Final presentation rehearsals
- ☐ Final presentation call (open and live streamed)
- ☐ Other:

Can you think of a topic that we did not share in our program with the cohort members and mentors that could be useful for Open Science projects and leaders?

Please share how you would define success for you as a mentor and for your mentee as an Open Science leader in the OLS program. *

Would you be happy for us to you publish your success statement from the previous question on the OLS website? *

Would you be interested in returning to the OLS-5 as a mentor or expert, and/or join our steering committee? *

__More about mentoring: https://openlifesci.org/about#mentors; more about being an expert: https://openlifesci.org/about#experts; more about our new steering committee: https://openlifesci.org/posts/2021/12/21/wt-open-research-fund/__

- ☐ Yes I'd like to return as a mentor
- ☐ Yes I'd like to return as an expert
- ☐ Yes I'd like to return as a collaborator to run this program in my network Yes I am interesting in joining the OLS steering committee
- ☐ I am not sure yet, but ask me later
- ☐ No, I would not be able to return to OLS-5
- ☐ Other:

What's your GitHub ID?

Optional, so we can re-use your profile from OLS-4 - see https://openlifesci.org/ols-4#mentors - Leave this field blank if you are taking a break from OLS-5. ALTERNATIVELY, if you want to update your profile, please fill out the full mentor registration form for OLS-5, here: [LINK REDACTED]

Would you recommend us to invite your mentee(s) to OLS-5? If you mentored a team of people who can be invited in different roles, please provide details in others. *

- ☐ Yes as a mentor
- ☐ Yes as an expert
- ☐ Yes, as a call facilitator
- ☐ No, they are not ready yet!
- ☐ Other:

If you would like us to invite someone else from your network as a mentor, mentee or expert in OLS-5, please provide their name and email followed by the recommended roles they will be interested in. *

Do you think your mentee was able to effectively engage with OLS throughout the program? *

We're asking this to get an idea of numbers since we noted a little drop-off in OLS-4. We're not 100% sure why but thought it might be due to pandemic-related fatigue, burnout, or illness, so we're exploring what options we can offer. Any info you can provide would be handy - we're also considering offering people the chance to return in OLS-5 if we have enough spots free. - [ ] Yes, they were able to engage - [ ] No, they had difficulty engaging or attending calls - [ ] Other:

Anything else you would like to share with us? (we have also created an anonymous survey: [LINK REDACTED] and encourage you to share any feedback, reflections and incident from the OLS-4 program that the OLS team should know.)
