# Supplementary material for: Ten simple rules for establishing a mentorship programme
Source: PLoS Comput Biol. 2022 May 12;18(5):e1010015. doi: 10.1371/journal.pcbi.1010015 (PMC9098017; doi:10.1371/journal.pcbi.1010015)
Supplement: S11 Text — The feedback form for one of the tracks of the ESCALATOR mentorship programme (EXPLORER). ESCALATOR used this feedback form to assess participation and evaluate effectiveness of this mentorship track. Similar forms will be developed for each of this programme’s tracks to assess success and impact. (PDF) [file pcbi.1010015.s011.pdf]

EXPLORER Feedback Form

Last updated: January 2022

Description: This is the feedback form for participants in the ESCALATOR Digital Champions Initiative EXPLORER track. For more information please visit our website - https://escalator.sadilar.org/champions/explorer/.

* Required

Registration and feedback for the EXPLORER track is optional, but by letting us know that you are working through the materials, we are able to support you better. By providing feedback you can help us to improve the programme and have a bigger impact on our community.

Thank you for providing feedback about the ESCALATOR Explorer Track. Your feedback will help shape and improve the experience for others.

1. *Email: ____________________

2. First name: ____________________

3. Surname: ____________________

4. *Have you watched any videos from the playlists available through the Explorer track? *(Select all playlists from which you have watched videos)*
   - ☐ What is Digital Scholarship?
   - ☐ What is Computational Thinking?
   - ☐ What is Digital Humanities?
   - ☐ What is Computational Social Sciences?
   - ☐ What is Open Access?
   - ☐ What is Open Research?
   - ☐ What are Open Educational Resources?
   - ☐ What is Reproducible Research?
   - ☐ What is Research Data Management?
   - ☐ Digital Humanities & Computational Social Sciences in Africa
   - ☐ I haven't watched any videos yet

5. *Have you participated in one of the monthly EXPLORER meetings? *You are welcome to join as many or few meetings as you want and can join them at any stage during your EXPLORER journey!)*
   - ☐ Yes
   - ☐ No

6. *Have you joined the community Slack workspace? *(More information available at https://escalator.sadilar.org/post/connect-with-the-community/)*
   - ☐ Yes
   - ☐ No

7. *How useful is the programme to help you get started with foundational concepts of digital scholarship in Digital Humanities or Computational Social Sciences?
   - ☐ 1 - Not at all useful
   - ☐ 2
   - ☐ 3
   - ☐ 4
   - ☐ 5 - Extremely useful

8. *How useful is the programme to help you connect to people from the Digital Humanities or Computational Social Science community in South Africa?
   - ☐ 1 - Not at all useful
   - ☐ 2
   - ☐ 3
   - ☐ 4
   - ☐ 5 - Extremely useful

9. *Which of the elements that form part of EXPLORER was the MOST useful to you?
   - ☐ Video playlists
   - ☐ Monthly introductory meeting
   - ☐ Networking with others
   - ☐ Joining the Slack workspace
   - ☐ Other: ________________________________

10. *Which of the elements that form part of EXPLORER was the LEAST useful to you?
    - ☐ Video playlists
    - ☐ Monthly introductory meeting
    - ☐ Networking with others
    - ☐ Joining the Slack workspace
    - ☐ Other: ________________________________

11. Please let us know if you can think of other topics that should be included in the curated playlists? *(You're welcome to include topic names, links to useful video resources or let us know if you'd like to create a video for a specific topic.)*

    ____
    ____

12. Can you recommend any good video resources for our existing topics or would you like to volunteer to create a video with a South African angle for any of the topics that we have already listed?

    ____
    ____

13. Do you have any questions about the programme?

    ____
    ____

14. Do you have any ideas how to improve the programme for newcomers to digital scholarship in Humanities or Social Sciences?

    ____
    ____

15. Do you have any other comments?

    ____
    ____

16. *Would you recommend the EXPLORER track to others wanting to get started with digital scholarship in South Africa?

    ____
    ____
